# Diabetic Atrial Cardiomyopathy: Pathogenesis, Diagnosis, Management, AI‐Driven Diagnosis, and Risk Prediction

**DOI:** 10.1155/jdr/4189404

**Published:** 2026-07-29

**Authors:** Helin Yang, Kexiao Yu, Bing Liang

**Affiliations:** ^1^ Chongqing Medical University-University of Leicester Joint Institute, Chongqing Medical University, Chongqing, China, cqmu.edu.cn; ^2^ Department of Pathology, College of Basic Medicine, Chongqing Medical University, Chongqing, China, cqmu.edu.cn; ^3^ Clinical Pathology Laboratory of Pathology Diagnostic Center, Chongqing Medical University, Chongqing, China, cqmu.edu.cn; ^4^ Molecular Medicine Diagnostic & Testing Center, Chongqing Medical University, Chongqing, China, cqmu.edu.cn; ^5^ Department of Orthopedics, Chongqing Traditional Chinese Medicine Hospital, The First Affiliated Hospital of Chongqing University of Chinese Medicine, Chongqing, China

**Keywords:** artificial intelligence, atrial fibrillation, atrial remodeling, diabetic atrial cardiomyopathy, diabetic cardiomyopathy

## Abstract

**Background:**

Diabetic atrial cardiomyopathy, a component of diabetic cardiomyopathy, is increasingly recognized. However, atrial‐focused mechanistic and clinical frameworks remain less developed than ventricular paradigms.

**Main Body:**

This review synthesizes the pathogenesis of diabetic atrial cardiomyopathy from conventional, multiomics, and translational perspectives. We highlight how diabetes‐related metabolic stress, inflammation, gut microbiota dysregulation, electrophysiological remodeling, genetic susceptibility, and epigenetic regulation converge on atrial fibrosis, conduction heterogeneity, contractile dysfunction, and thrombogenicity. These processes increase susceptibility to atrial fibrillation, heart failure, and embolic events. We integrate diagnostic strategies, including electrocardiographic indices, biomarkers, and multimodality imaging, with emphasis on left atrial size, strain, and fibrosis assessment. We also appraise emerging artificial intelligence approaches using electrocardiograms, imaging, and wearable signals while emphasizing that most models are not yet validated in DAtCM‐specific cohorts. Finally, we outline an integrated management framework that combines cardiometabolic optimization, lifestyle and rehabilitation strategies, guideline‐directed anticoagulation when indicated, and cautious development of upstream disease‐modifying interventions.

**Conclusion:**

Integrating mechanistic, multiomics, and artificial intelligence–enabled approaches may improve early identification and risk stratification of diabetic atrial cardiomyopathy. Dedicated atrial phenotyping cohorts and prospective trials are needed before these concepts can be translated into routine care.

## 1. Introduction

Diabetes mellitus (DM) is a common chronic metabolic disease that causes multiorgan complications. The global burden of diabetes continues to rise, with projections suggesting that hundreds of millions of adults will be affected in the coming decades [1, 2]. DM is strongly associated with cardiovascular disease (CVD) and is an important contributor to cardiomyopathy [3, 4]. Diabetic cardiomyopathy refers to diabetes‐related structural and functional myocardial abnormalities that can lead to cardiac dysfunction and heart failure (HF). Most mechanistic and clinical work has focused on ventricular remodeling [5]. By contrast, the effects of diabetes on atrial structure, electrophysiology, and function remain less systematically defined. Atrial cardiomyopathy (AtCM) is defined as any complex of structural, architectural, contractile, or electrophysiological changes affecting the atria with the potential to produce clinically relevant manifestations [6].

Recent advances in genomics, epigenomics, metabolomics, microbiome science, and artificial intelligence (AI) have created new opportunities to understand, detect, and manage diabetic atrial cardiomyopathy (DAtCM). These developments are clinically relevant because diabetes promotes atrial remodeling, atrial fibrillation (AF), HF, and thromboembolic risk through intertwined metabolic, inflammatory, microvascular, and electrophysiological pathways. This review summarizes conventional and multiomics mechanisms of DAtCM, evaluates diagnostic and risk‐prediction approaches, and discusses cardiometabolic and upstream therapeutic strategies. Distinct from previous reviews of diabetic cardiomyopathy, which have largely centered on ventricular disease, we frame DAtCM as an atrial disease entity and explicitly link diabetes‐induced atrial remodeling to AF and HF risk. We also outline a pragmatic AI‐enabled multimodal pathway for early detection and risk stratification while emphasizing the current need for DAtCM‐specific validation cohorts.

## 2. Search Strategy and Selection Criteria

Data for this review were identified by searches of PubMed, Web of Science, and references from relevant articles, guidelines, and reviews using the search terms “Diabetes AND Cardiomyopathy,” “Diabetes AND Atrial remodeling,” “Diabetes AND Atrial fibrillation,” “Atrial cardiomyopathy,” and “Artificial intelligence AND Diabetic cardiomyopathy.” Only peer‐reviewed publications published in English between 1996 and 2025 were included.

## 3. Classification, Consequences, and Diagnosis of AtCM

Because DAtCM is conceptualized as an AtCM with a specific DM background, a comprehensive understanding of the histopathological classification, clinical consequences, and detection strategies of general AtCM is essential for defining and detecting DAtCM in both clinical practice and research contexts.

### 3.1. Classification of AtCM

**Figure 1:**
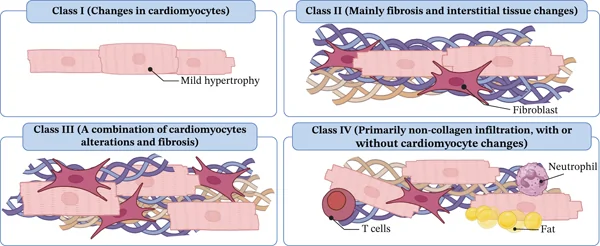
The histological and pathophysiological classification of atrial cardiomyopathy. Class I: Principal cardiomyocyte changes, including cardiomyocyte hypertrophy and myocytolysis, with minimal fibrosis. Class II: Principally fibrotic changes, with interstitial fibrosis predominating over cardiomyocyte alterations. Class III: Combined cardiomyocyte pathology and fibrosis, characterized by cardiomyocyte alterations together with collagen fiber deposition. Class IV: Primarily noncollagen interstitial infiltration or deposition, including amyloid deposition, inflammatory cell infiltration, fatty infiltration, and other noncollagen interstitial changes. Created with http://biorender.com/.

**Figure 2:**
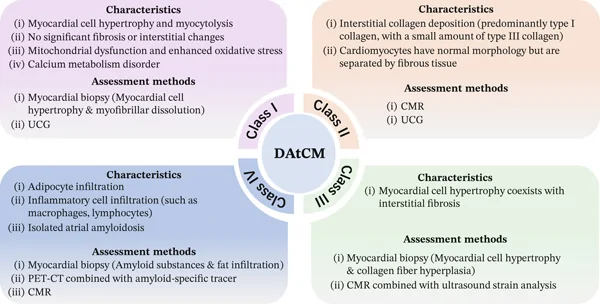
Characteristics and assessment methods for each EHRAS class. UCG, ultrasonic cardiogram; CMR, cardiac magnetic resonance; PET‐CT, positron emission tomography–computed tomography.

AtCM is classified by histological and pathophysiological features into four EHRAS classes (Figure 1): (I) principal cardiomyocyte changes, (II) principally fibrotic changes, (III) combined cardiomyocyte pathology and fibrosis, and (IV) primarily noncollagen infiltration, with or without cardiomyocyte changes. This classification was proposed by the European Heart Rhythm Association, Heart Rhythm Society, Asian Pacific Heart Rhythm Society, and Sociedad Latino Americana de Estimulacion Cardiaca y Electrofisiologia, and it is not intended to describe a linear disease progression [6]. The corresponding characteristics and assessment methods are summarized separately in Figure 2.

Class I primarily involves cardiomyocyte changes, characterized mainly by cardiomyocyte hypertrophy and myocytolysis, without prominent interstitial fibrosis or other major interstitial pathological changes [6]. Genetic and stress‐responsive signaling are important contributors. Under pressure overload or other pathological conditions, cardiomyocytes activate transcription factors such as nuclear factor of activated T cells (NFAT), leading to the expression of hypertrophy‐related genes [7]. Genetic and metabolic alterations may also be associated with metabolic reprogramming, including an altered balance between fatty acid and glucose utilization, which may reflect an adaptive response to stress but can become maladaptive over time [8]. Electrophysiological remodeling is reflected by altered L‐type calcium channel and potassium channel expression or function. Calcium signaling contributes to hypertrophic growth through Calcineurin A–mediated NFAT dephosphorylation and nuclear translocation, with increased expression of hypertrophy‐related markers such as atrial natriuretic peptide (ANP) and *β*‐myosin heavy chain [7, 9–11]. Ion channel and calcium‐handling remodeling may disrupt depolarization and repolarization, promote calcium overload and cellular stress, and thereby contribute to cardiomyocyte dysfunction and electrical instability. Early afterdepolarizations (EADs) and delayed afterdepolarizations (DADs) are also relevant triggers of arrhythmogenic activity [12, 13]. EADs occur during repolarization and are favored by reduced repolarizing potassium currents or sustained calcium influx. These changes prolong action potential duration and can trigger premature atrial activity. DADs occur after repolarization, usually in the setting of sarcoplasmic reticulum calcium overload and spontaneous calcium release. The resulting inward current, often mediated by the sodium–calcium exchange, can promote triggered activity and arrhythmogenesis [12].

Class II is characterized by relatively preserved cardiomyocyte appearance with predominant fibrosis and interstitial remodeling. Aging, cigarette smoking, hypertension, and AF are major associated factors [6]. Fibrotic remodeling disrupts cell‐to‐cell coupling, increases conduction heterogeneity, and provides an arrhythmogenic substrate that facilitates AF initiation and maintenance [14, 15]. The interstitium remodels through extracellular matrix accumulation, especially collagen deposition between myocytes. Aging, oxidative stress, and inflammation can activate transforming growth factor‐beta (TGF‐*β*) and Angiotensin II (Ang II), which are important but not exclusive profibrotic drivers [16, 17]. TGF‐*β* promotes cardiac fibroblast activation, collagen synthesis, and extracellular matrix accumulation through canonical SMAD signaling and non‐SMAD pathways, including mitogen‐activated protein kinase (MAPK) and PI3K‐AKT signaling [18, 19]. Ang II further amplifies fibrotic remodeling through AT1 receptor–mediated oxidative stress and ACE/ERK‐associated fibroblast or interstitial cell activation [16, 20].

Class III is characterized by a combination of cardiomyocyte abnormalities and fibrotic changes. HF and diabetes are important contributing conditions, particularly in patients with diabetes‐related microvascular dysfunction. In HF, chronic pressure or volume overload and neurohormonal activation can promote atrial stretch, extracellular matrix accumulation, and fibrosis. Advanced glycation end product (AGE) formation is increased by chronic hyperglycemia in diabetes. AGEs can signal largely through the receptor for AGEs (RAGE), thereby initiating oxidative stress, inflammatory cascades, and profibrotic pathways [21]. Alterations in calcium transients, calcium‐handling proteins, and atrial tissue architecture can further disrupt calcium cycling and excitation–contraction coupling [22]. Autonomic remodeling is another important process. Regional heterogeneity in sympathetic and parasympathetic innervation within remodeled atrial tissue may contribute to sympathovagal imbalance and promote AF [23]. Together, atrial stiffness, endocardial dysfunction, local inflammation, thrombogenicity, slowed conduction, and ectopic activity increase susceptibility to AF [24].

Class IV is characterized primarily by noncollagen interstitial infiltration, including amyloid deposition, cellular inflammation, adipose infiltration, and other interstitial changes [6]. Atrial amyloidosis may arise from abnormal aggregation of amyloidogenic proteins, including ANP‐derived deposits, within atrial tissue. Once nucleation occurs, fibril growth can be accelerated or restrained by local molecular cofactors and by changes in cardiac or extracardiac stimuli. Amyloid deposition between atrial endothelial cells and cardiomyocytes can provoke inflammation, fibrosis, and disruption of myocardial architecture [25]. Inflammatory activation is also closely linked to AF. Circulating and tissue inflammatory mediators, including C‐reactive protein, tumor necrosis factor, and interleukins, can promote endothelial activation, platelet activation, fibroblast signaling, and atrial remodeling [26]. Adipose infiltration, particularly in the setting of aging and obesity, may further disturb myocardial metabolism, conduction, and mechanical function [27]. These processes can coexist and reinforce one another, producing a complex atrial substrate that favors structural remodeling, functional impairment, and arrhythmogenesis.

### 3.2. Clinical Consequences of AtCM

The four EHRAS classes share clinical consequences, including AF, other atrial arrhythmias, stroke, and sudden cardiac death. In Class I disease, electrophysiological changes increase atrial arrhythmia risk through electrical remodeling and altered excitability [6, 7, 21]. In Class II disease, fibrotic and interstitial remodeling commonly coexist with left atrial (LA) enlargement, HF risk, and AF susceptibility [6, 14]. In Classes III and IV, vascular injury, endocardial dysfunction, and atrial stasis can increase thromboembolic risk [6]. A recent EHRA/HRS/APHRS/LAHRS consensus further frames AtCM as mild, moderate, or severe according to the degree of atrial functional impairment detected by clinical examination [28].

### 3.3. Diagnosis of AtCM

Accurate diagnosis and stage‐appropriate treatment require layered assessment of atrial structure, function, electrophysiology, and circulating biomarkers.

Echocardiography is the preferred screening and follow‐up method for LA disease because it provides reproducible measurements of LA size and function. The commonly used upper normal indexed LA volume is 34 mL/m^2^ in adults, based on ASE/EACVI chamber quantification and diastolic function recommendations; interpretation should still consider body size, rhythm, and loading conditions [29, 30]. Three‐dimensional echocardiography can improve volume accuracy and reproducibility compared with two‐dimensional imaging, particularly when image quality is adequate [31].

Doppler echocardiography can complement LA volumetric assessment by characterizing transmitral inflow and the atrial contribution to ventricular filling. Age‐stratified reference values reported by Nikitin et al. include the following peak *E*‐wave velocity, peak *A*‐wave velocity, and *E*/*A* ratios [32]:

• 20–39 years: *E* = 0.76 m/s, *A* = 0.65 m/s, and *E*/*A* = 1.3
• 40–59 years: *E* = 0.63 m/s, *A* = 0.56 m/s, and *E*/*A* = 1.2
• 60–79 years: *E* = 0.76 m/s, *A* = 0.63 m/s, and *E*/*A* = 1.4
• 80 years or older: *E* = 0.77 m/s, *A* = 0.71 m/s, and *E*/*A* = 1.3

The third modality is strain and strain rate imaging. Reported normal values for LA systolic, early diastolic, and late diastolic strain rates include 3.4 ± 1.0, 3.9 ± 1.7, and −3.1 ± 1.0 s^−1^, respectively [33]. Cardiac computed tomography can quantify atrial volume and detect LA appendage thrombus with high sensitivity and specificity in AF cohorts, although its positive predictive value varies with pretest probability [34]. Cardiac magnetic resonance imaging (CMRI) is less available and more expensive than echocardiography, but late gadolinium enhancement CMRI can detect atrial fibrosis and may be useful in research settings and selected clinical scenarios [35, 36].

Electrocardiography (ECG) is a readily available tool for evaluating atrial electrical remodeling. Although ECG findings alone are not diagnostic, the following parameters may suggest AtCM when interpreted alongside imaging and clinical context:

1. P‐wave duration and interatrial block

P‐wave duration ≥ 120 ms indicates partial interatrial block; biphasic P waves in Leads II, III, and aVF indicate advanced interatrial block [28].

2. Amplified digital P‐wave duration

Amplified digital P‐wave duration, measured from amplified digital ECG recordings, may reflect AtCM severity: 140–150 ms indicates a discrete stage, 150–180 ms indicates a moderate stage, and > 180 ms or positive–negative P waves in two of three inferior leads indicates an advanced stage [28].

3. P‐wave terminal force in Lead V1

PTFV1 is calculated from the duration and absolute amplitude of the terminal negative P‐wave component in V1; an absolute value > 4 mV·ms is considered pathological [28].

4. P‐wave axis

A frontal P‐wave axis outside 0° to +75° is abnormal and is associated with future AF detection [28].

5. P‐wave voltage

P‐wave voltage ≤ 0.1 mV in Lead I is considered abnormal and has been associated with AF onset or recurrence [28].

Compared with imaging, circulating biomarkers are more accessible and may support the diagnosis of cardiac injury, dysfunction, or atrial remodeling when interpreted with ECG and imaging findings. Cardiac troponins, including cardiac troponin I (TnI), high‐sensitivity cardiac troponin T (hs‐cTnT), and high‐sensitivity cardiac troponin I (hs‐cTnI), reflect myocardial injury when detected in serum. However, high‐sensitivity troponin immunoassays can be affected by heterophile antibodies, autoantibodies, or macrotroponin complexes, which may cause false‐positive or assay‐dependent results. *N*‐terminal pro‐B‐type natriuretic peptide (NT‐proBNP) is also an important biomarker of cardiac wall stress, ventricular dysfunction, and volume or pressure overload [37, 38]. Other natriuretic peptides, including brain natriuretic peptide and ANP, may provide complementary information about myocardial stress and chamber remodeling. Additional biomarkers, including matrix metalloproteinases, tissue inhibitors of metalloproteinases, IL‐33, and soluble suppression of Tumorigenicity 2, remain promising but require DAtCM‐specific validation [38].

The EHRAS classes have distinct but overlapping arrhythmic and structural consequences. Diagnosis requires layered assessment, including ECG indices such as P‐wave duration and voltage, advanced imaging such as strain and volumetric analysis, and biomarkers such as troponin and NT‐proBNP. These assessment approaches are summarized in Figure 2. Tailored management should target the dominant substrate, including fibrosis, calcium dysregulation, infiltration, metabolic injury, or thrombogenicity.

## 4. Pathophysiology of DAtCM

**Figure 3:**
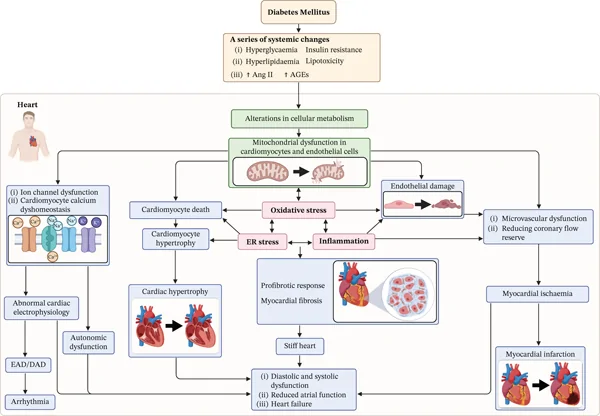
Mechanism of diabetic atrial cardiomyopathy. Ang II, Angiotensin II; AGEs, advanced glycation end products; ER, endoplasmic reticulum; EAD, early afterdepolarization; DAD, delayed afterdepolarization. Created with http://biorender.com/.

DM induces electrophysiological, structural, systolic and diastolic, metabolic, hypercoagulable, autonomic, and microvascular alterations that promote AtCM. This sequence follows the organization of the sections below. Diabetes‐related myocardial fibrosis, collagen accumulation, and increased atrial stiffness disrupt tissue architecture. Metabolic disorders, mitochondrial dysfunction, and chronic hyperglycemia compromise energy handling and electrical stability. Microvascular injury promotes ischemia, inflammation, and interstitial fibrosis. Together, these processes reduce atrial function, slow conduction, and create a substrate for DAtCM (Figure 3) [28, 39].

### 4.1. Electrophysiological Alterations in the Atrium Induced by DM

Patients with diabetes can exhibit impaired intracellular calcium handling and altered ion channel function, particularly in cardiomyocytes affected by diabetic heart disease [40].

Disturbed intracellular calcium homeostasis is closely related to impaired sarcoplasmic reticulum function [40]. Diabetes‐associated oxidative stress can damage the sarcoplasmic reticulum and reduce calcium storage capacity. Metabolic stress, oxidative injury, and posttranslational modifications affect L‐type calcium channels, Ryanodine Receptor Type 2 (RyR2), sodium–calcium exchanger (NCX), and sarco/endoplasmic reticulum calcium‐ATPase (SERCA). These abnormalities disrupt calcium cycling, impair contractility, and increase susceptibility to atrial arrhythmia [41].

#### 4.1.1. L‐Type Calcium Channel

In diabetic models with insulin resistance (e.g., Type 2 diabetes db/db mice), L‐type calcium channel expression is reduced, diminishing calcium influx and impairing calcium‐induced calcium release from the SR. However, shifts in steady‐state inactivation enhance the “window current,” increasing the likelihood of EAD, which heightens the risk of arrhythmia [41].

#### 4.1.2. RyR2

Diabetes can induce maladaptive RyR2 remodeling and increase diastolic sarcoplasmic reticulum Ca^2+^ leak [41]. In streptozotocin Type 1 diabetic rats and high‐sucrose diet metabolic syndrome rats, reported RyR2 alterations include increased Ser2808 phosphorylation, reduced FK506‐binding protein 12.6 binding, and enhanced sarcoplasmic reticulum Ca^2+^ leak [41]. Ser2808 phosphorylation was also increased in high‐fat diet–fed dogs, whereas RyR2 dysfunction in lipid overload and high‐fat diet obesity mouse models has been linked more closely to oxidative modification [41]. For Ser2814, studies in streptozotocin diabetic rats and db/db mice suggest that Calcium/Calmodulin‐Dependent Kinase II (CaMKII) may contribute to RyR2 remodeling, but direct evidence showing phosphorylation of RyR2 at Ser2814 by CaMKII in db/db mice remains limited [41]. Recent atrial studies provide further support for this pathway. In streptozotocin Type 1 diabetes and high‐fat diet plus low‐dose streptozotocin Type 2 diabetes mouse models, CaMKII contributed to increased AF susceptibility [42]. In long‐term high‐fat diet Type 2 diabetic mice, increased phosphorylation of atrial RyR2 at Ser2814 and RyR2 Ca^2+^ leak have also been reported [43]. These alterations can promote Ca^2+^ sparks and waves, activate NCX, and generate DADs, thereby increasing arrhythmia susceptibility [41, 43].

#### 4.1.3. NCX

NCX links intracellular calcium handling to membrane depolarization by extruding one Ca^2+^ ion in exchange for three Na^+^ ions. In diabetic myocardium, altered NCX expression or activity may either compensate for impaired sarcoplasmic reticulum calcium reuptake or promote arrhythmogenic inward current during spontaneous calcium release. Because reported NCX changes vary by diabetes model, chamber, disease duration, and metabolic state, their role in DAtCM should be interpreted as context‐dependent rather than uniformly protective or harmful [41].

#### 4.1.4. SERCA

SERCA2a expression and activity are downregulated in diabetes, mainly due to decreased mRNA expression and oxidative modifications. This impairs calcium reuptake by the SR, reducing calcium transient amplitude, slowing myocardial relaxation, and promoting cytoplasmic calcium overload, which contributes to atrial alternans and increases susceptibility to atrial arrhythmias [41].

High glucose promotes oxidative stress, intracellular calcium overload, and activation of the local renin–angiotensin system. Ang II, acting through Ang II Type 1 receptors, stimulates NADPH oxidase–derived reactive oxygen species (ROS). ROS and calcium‐dependent signals activate nuclear factor‐*κ*B (NF‐*κ*B), which can further regulate renin–angiotensin system–related genes and oxidative stress pathways, thereby sustaining an amplification loop. Activated NF‐*κ*B can bind the promoter of SCN5A and inhibit transcription, reducing Nav1.5 expression. This impairment of the cardiac sodium current slows rapid depolarization, reduces conduction stability, and increases arrhythmogenic vulnerability [44, 45].

Potassium channel remodeling in DM is complex. Hyperglycemia, oxidative stress, inflammation, and electrolyte imbalance can reduce repolarizing potassium currents and prolong action potential duration. TWIK‐Related Acid‐Sensitive Potassium Channel 1 (TASK‐1) is a pH‐sensitive potassium channel, and extracellular acidification can inhibit TASK‐1 outward current, thereby potentially affecting cardiac repolarization. However, direct evidence that diabetic intracellular acidosis reduces TASK‐1 current in atrial cardiomyocytes remains limited, and the cited TASK‐1 studies are not all diabetes‐specific [40, 46]. Therefore, TASK‐1 should be considered a plausible mechanistic link requiring direct validation in DAtCM models.

### 4.2. Atrial Structure Remodeling Induced by DM

DM induces structural remodeling in the atrium, encompassing atrial hypertrophy, myocardial fibrosis, myocyte disarray, and architectural disorganization [47, 48].

In a high‐fat diet plus low‐dose streptozotocin model of diabetes, mice developed atrial hypertrophy, heavier atria, widened interstitial spaces, myocyte disarray, and collagen deposition. These morphological changes were associated with activation of the Poly (ADP‐Ribose) Polymerase‐1 (PARP‐1), I*κ*B kinase *α* (IKK*α*), and NF‐*κ*B axis in atrial tissue, suggesting that metabolic injury can induce atrial wall remodeling in experimental models [48].

Mechanistically, chronic hyperglycemia and dyslipidemia impose oxidative and genotoxic stress on atrial cardiomyocytes. ROS‐induced DNA damage activates PARP‐1, which synthesizes poly (ADP‐ribose) chains and helps assemble a nuclear signaling complex involving IKK*α*. This promotes NF‐*κ*B nuclear translocation and sustains a proinflammatory, growth‐promoting atrial milieu. PARP‐1 activity also consumes nicotinamide adenine dinucleotide (NAD+), which can worsen energetic stress and inflammatory amplification [48]. Because most validation data for this axis derive from animal or cellular models, its activity in human diabetic atrial tissue requires further confirmation.

Interstitial and replacement fibrosis are major structural features of diabetic atrial remodeling. Upstream PARP‐1 activation and IKK*α*/NF‐*κ*B signaling, superimposed on systemic metabolic stress, can prime fibroblast activation and extracellular matrix accumulation. NF‐*κ*B‐dependent inflammatory mediators, including TNF‐*α* and IL‐1*β*, amplify fibroblast proliferation, collagen synthesis, and NLRP3 inflammasome activation [48]. Hyperglycemia also promotes AGE accumulation; AGE‐RAGE signaling can activate TGF‐*β*1/SMAD signaling and may promote Wnt/*β*‐catenin activation, thereby contributing to myofibroblast transformation and collagen deposition [49, 50]. Downregulation of microRNAs such as miR‐133a may increase profibrotic gene expression, including connective tissue growth factor (CTGF), thereby reinforcing TGF‐*β*1‐driven matrix remodeling [51].

Myocyte disarray and architectural disorganization are integral to this remodeling continuum. Mitochondrial dysfunction and disturbed redox balance in diabetic hearts destabilize cytoskeletal and sarcomeric structure, while PARP‐1‐driven NAD+ depletion may impair energy‐dependent maintenance of myofibrillar organization. Concurrent NF‐*κ*B activation may modify the expression of structural and stress‐response proteins, further disturbing cell alignment and tissue architecture. The result is the broadened interstitial spaces and disordered myocyte arrangement seen on histology [48].

### 4.3. Atrial Systolic and Diastolic Dysfunction

The effects of DM on cardiac troponin, myosin, intracellular calcium regulation, and other factors result in atrial contraction and relaxation disorders [39].

In the study by Zhang et al., the *E*/*E* ^′^ ratio of diabetic mice was significantly increased, measuring 34.53 ± 1.73 in the T2DM group and 25.39 ± 1.24 in the control group at 24 weeks. Furthermore, the *E*/*A* ratio in the diabetic mice was significantly lower compared to that in the control group at 12, 16, and 24 weeks [52].

The affected troponins encompass TnI, troponin T (TnT), and troponin C (TnC). The primary factors influencing their function are alterations in phosphorylation and structural modifications. These changes ultimately result in diminished calcium binding affinity and reduced myocardial calcium sensitivity [39].

There was an alteration in the phosphorylation status of TnI, predominantly characterized by an increased level of phosphorylation. Consequently, this modification influenced its calcium‐binding affinity, leading to a reduced sensitivity of the myocardium to calcium and thereby affecting the contractile function of the myocardium [53]. Phosphorylation of TnI reduces myofilament Ca^2+^ sensitivity, thereby decreasing the responsiveness of muscle contraction to calcium [54]. Studies have demonstrated that in diabetic cardiomyopathy, TnI expression may be diminished, often in conjunction with apoptosis or dysfunction of cardiomyocytes [39].

Heart‐specific TnT expression may be diminished in diabetic myocardium. This downregulation could lead to decreased sensitivity of cardiac muscle to calcium, consequently impairing myocardial contractile function. Additionally, the phosphorylation level of TnT is also impacted by DM, typically exhibiting a reduction [53]. Furthermore, the structure of TnT may be modified through glycation or other posttranslational modifications. Such alterations could potentially impact the interaction between TnT and other calcium‐regulatory proteins, including TnC and actin, thereby influencing myocardial contraction [53]. Owing to alterations in TnT, there may be a significant reduction in the calcium sensitivity of cardiomyocytes, leading to an inability of the myocardium to generate adequate contractile force at the same calcium concentration [39].

TnC is similarly affected in a manner analogous to TnT, with its phosphorylation status also being reduced [39]. TnC undergoes glycation in the presence of hyperglycemia. This posttranslational modification alters its three‐dimensional structure and functionality, potentially disrupting the role of cardiomyocytes in calcium signaling transduction [53].

In addition to troponin, the impaired Ca^2+^ handling in cardiomyocytes, as discussed earlier in relation to electrophysiological alterations associated with diabetes, also promotes the development of cardiac diastolic dysfunction [39].

### 4.4. Metabolic Factors Associated With AtCM in the Context of DM

DM induces metabolic alterations in the heart, primarily manifested in three key areas: enhanced fatty acid metabolism, reduced glucose metabolism, and modifications in energy production. These changes lead to cardiac dysfunction, inadequate energy supply to the myocardium, cardiac remodeling, and other associated issues.

#### 4.4.1. Enhanced Fatty Acid Metabolism

In diabetic myocardium, insulin resistance reduces glucose uptake and favors greater reliance on fatty acid uptake and oxidation [55, 56]. In Type 2 diabetes, insulin resistance and increased plasma fatty acid concentrations can shift myocardial substrate use from glucose toward fatty acids, creating an imbalance in which fatty acid uptake exceeds oxidative capacity and thereby contributes to lipotoxicity [57]. Elevated free fatty acids (FFAs) and activation of lipid metabolic transcriptional regulators, especially peroxisome proliferator–activated receptor *α* (PPAR*α*), can increase fatty acid oxidation in some settings [57]. However, PPAR*α* expression and activity in diabetic hearts are not uniform across models or disease stages. Some studies suggest activation of the PPAR*α* pathway, whereas others show PPAR*α* downregulation or impaired signaling, particularly when lipotoxicity, mitochondrial dysfunction, and HF are present [58]. This controversy supports a balanced interpretation: Altered PPAR*α* signaling reflects metabolic remodeling, but its direction and consequences depend on diabetes type, duration, and treatment context.

#### 4.4.2. Decreased Glucose Metabolism

In comparison to the enhanced FFA metabolism, a reduction in cardiac glucose metabolism also represents a significant characteristic [56]. Insulin resistance results in a diminished responsiveness of cardiomyocytes to insulin. This condition inhibits the insulin signaling pathway, consequently suppressing the expression and activity of glucose transporters like Glucose Transporter‐4, thereby reducing glucose uptake and utilization by cardiomyocytes [57]. Alterations in the adenosine monophosphate–activated protein kinase (AMPK) signaling pathway, which normally functions as a critical energy‐sensing enzyme that regulates cellular metabolism in response to energy stress, have been observed. In DM, the activity of AMPK is suppressed, resulting in increased fatty acid oxidation and decreased glucose oxidation [55].

DM induces a shift in energy production in cardiac cells, transitioning from a reliance on glucose metabolism to a greater dependence on fatty acid oxidation. This metabolic reprogramming is characterized by decreased glucose utilization and enhanced fatty acid metabolism [55].

The direct impact of DM on myocardial metabolism is primarily manifested in the alteration of energy production pathways. Specifically, the reduced metabolic reliance of the myocardium on proximal energy substrates can impair cardiac pumping function, potentially leading to symptoms such as HF [55]. Concurrently, the reduced utilization of glucose may result in diminished ATP synthesis within cardiomyocytes, which in turn can impair myocardial contractility [56].

Lipotoxicity in the diabetic heart, induced by an excess of fatty acids, primarily affects cardiac function through lipid accumulation. This accumulation triggers an inflammatory response and subsequently promotes oxidative stress, ultimately leading to myocardial cell dysfunction. Elevated intracellular lipid levels and high concentrations of FFAs within cardiomyocytes impair mitochondrial function [55–57].

### 4.5. Diabetic Hypercoagulable State

In diabetes, a procoagulant milieu pertinent to DAtCM is characterized by excessive activation of coagulation factors and impaired fibrinolysis, with higher plasma fibrinogen, tissue factor pathway inhibitor, thrombin–antithrombin complex, and Plasminogen Activator Inhibitor‐1 [59]. Nonenzymatic glycation undermines anticoagulant control (e.g., glycation of Antithrombin III weakens its heparin‐mediated activity) [60]. Platelets are hyperreactive, hyperglycemia‐driven membrane glycation reduces membrane fluidity and increases calcium influx, adhesion molecule expression rises (P‐selectin, tissue factor, and von Willebrand factor), and responsiveness to nitric oxide and prostacyclin is blunted; concomitant endothelial dysfunction further diminishes these anticoagulant signals [59, 61]. Glycation of fibrin and accumulation of AGEs decrease susceptibility to fibrinolysis, while oxidative stress, inflammation, and endothelial glycation enhance tissue factor expression and stabilize fibrin [59, 61].

Hypercoagulability may promote AtCM because thrombin and factor Xa activate protease‐activated receptors on endothelial cells and fibroblasts, triggering inflammation and profibrotic signaling. In primary adult human atrial cardiac fibroblasts, factor Xa upregulates profibrotic genes, including *α*‐smooth muscle actin and TGF‐*β*, as well as inflammatory mediators such as IL‐6 and C‐C Motif Chemokine Ligand 2, mainly through Protease‐Activated Receptor‐1. In experimental models, factor Xa inhibition attenuates atrial endomysial fibrosis and may reduce atrial myocyte hypertrophy, supporting a mechanistic contribution of coagulation to atrial remodeling. However, current evidence largely derives from experimental models and in vitro studies, and its clinical relevance and translational value in DAtCM require prospective validation [62].

### 4.6. Cardiac Autonomic Neuropathy (CAN) Induced by DM

DM can cause autonomic neuropathy characterized by persistent sympathetic activation and reduced vagal activity [39]. Insulin resistance and chronic hyperinsulinemia activate the renin–angiotensin–aldosterone system, while Ang II enhances norepinephrine release, and aldosterone contributes to vasoconstriction, myocardial stiffness, and sustained sympathetic drive [63]. Hyperglycemia may upregulate *β*1‐adrenergic receptors and bias signaling toward MAPK and Protein Kinase A pathways, whereas oxidative stress impairs insulin signaling and Glucose Transporter‐4 translocation [63, 64]. Sympathetic predominance promotes cardiomyocyte hypertrophy, interstitial fibrosis, and inflammatory activation, including M1 macrophage polarization, TNF‐*α* release, and TGF‐*β*1‐SMAD signaling. In parallel, AGEs, oxidative stress, inflammation, and insulin resistance can impair vagal structure and muscarinic signaling. The combined autonomic imbalance fosters atrial stiffness, fibrosis, and inflammatory amplification [63–65].

### 4.7. Myocardial Microvascular Dysfunction in DAtCM

**Figure 4:**
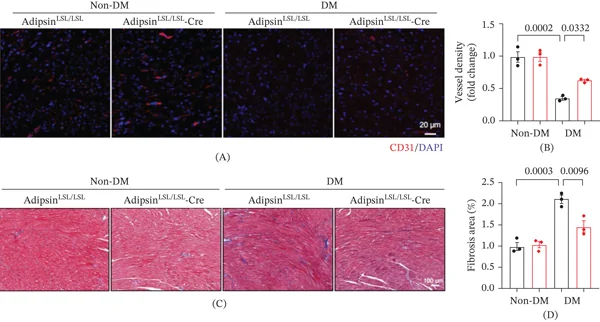
Cardiac microvascular injury and myocardial fibrosis in diabetic cardiomyopathy models. (A) Representative CD31/DAPI immunofluorescence images of cardiac tissue from the experimental groups in the original study. Scale bar = 20 * μ*m. (B) Quantification of vessel density based on CD31 immunofluorescence. (C) Representative Masson trichrome staining of cardiac tissue. Scale bar = 100 * μ*m. (D) Quantification of fibrosis area. In this review, these panels are used to illustrate diabetes‐associated cardiac microvascular rarefaction and myocardial fibrosis. Adipsin‐related intervention groups from the original figure are retained for completeness. Adapted from Ref. [67] under the CC BY 4.0 license. Copyright 2023, the authors.

**Figure 5:**
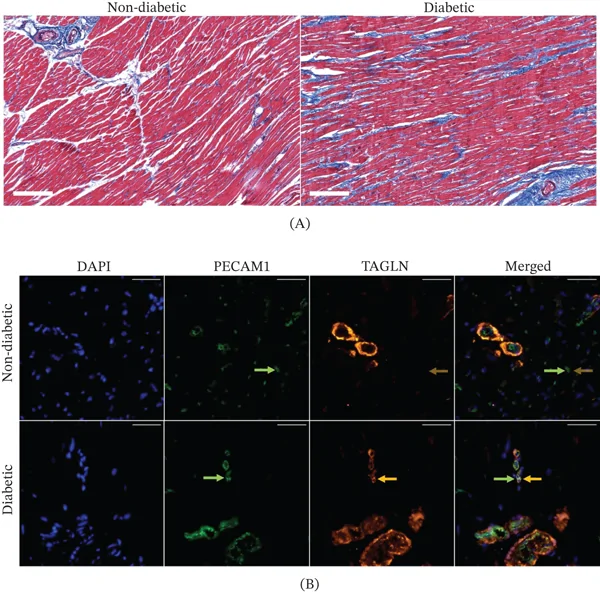
Human evidence of cardiac fibrosis and EndMT in diabetic cardiomyopathy. (A) Representative trichrome staining of cardiac tissue from nondiabetic individuals and patients with diabetes, showing increased perivascular and interstitial fibrosis in diabetic hearts. (B) Representative immunofluorescence staining for DAPI, PECAM1/CD31, and TAGLN/SM22 in small cardiac vessels. The merged images show PECAM1/TAGLN colocalization, supporting diabetes‐associated endothelial‐to‐mesenchymal transition in the heart. DAPI, 4 ^′^,6‐diamidino‐2‐phenylindole; PECAM1, Platelet and Endothelial Cell Adhesion Molecule 1; TAGLN, transgelin. Adapted from Ref. [69] under the CC BY 4.0 license. Copyright 2023, the authors.

DM induces cardiac microcirculatory dysfunction through multiple mechanisms, contributing to myocardial ischemia and interstitial fibrosis. Endothelial cell–derived Endothelin‐1 (ET‐1) has been implicated in diabetic cardiac fibrosis by upregulating TGF‐*β* and CTGF expression; activating downstream TGF‐*β* signaling, including Akt activation and Snail stabilization; and promoting VE‐cadherin loss and endothelial‐to‐mesenchymal transition (EndMT) [66]. In diabetic cardiomyopathy models, cardiac microvascular injury is characterized by impaired coronary microvascular integrity, reduced CD31‐positive microvascular density, and myocardial fibrosis (Figure 4) [67]. Coronary flow reserve (CFR) is often reduced in DM through multiple mechanisms [68]. Reduced CFR may promote atrial ischemia, oxidative stress, and inflammation, thereby destabilizing myocardial electrical activity and indirectly promoting AF. Human cardiac tissue from patients with diabetic cardiomyopathy shows increased perivascular and interstitial fibrosis, reduced endothelial marker expression, and CD31/SM22 colocalization in small vessels, supporting diabetes‐associated EndMT in the heart (Figure 5) [69]. In summary, diabetes reduces myocardial blood supply and promotes interstitial fibrosis, thereby contributing to the initiation and progression of DAtCM.

## 5. Investigating the Association Between DM and AtCM From a Multiomics Perspective

**Figure 6:**
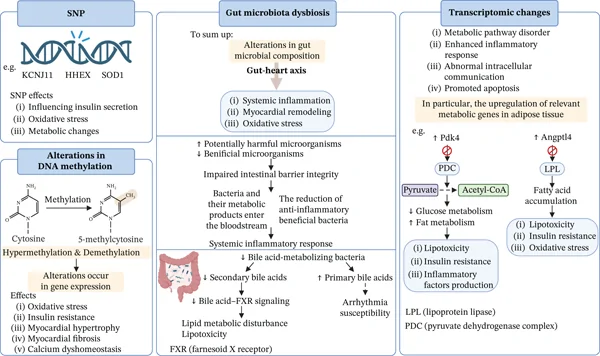
The impact of diabetes on the heart from a multiomics perspective. SNP, single‐nucleotide polymorphism; FXR, farnesoid X receptor; LPL, lipoprotein lipase; PDC, pyruvate dehydrogenase complex; PDK4, Pyruvate Dehydrogenase Kinase 4; ANGPTL4, Angiopoietin‐Like 4; DAtCM, diabetic atrial cardiomyopathy. The genetic associations listed here should be interpreted as candidate links to diabetes‐related metabolic and oxidative pathways, rather than as established DAtCM‐specific causal loci. The relationship between bile acid–FXR signaling and arrhythmia susceptibility is presented as indirect and associative and requires further validation. Created with http://biorender.com/.

Multiomics approaches focusing on single‐nucleotide polymorphism (SNP), epigenetics, transcriptomics, and gut microbiota are crucial for understanding DAtCM, as they provide a new perspective for elucidating how diabetes‐related genetic and regulatory factors translate into atrial remodeling and increased vulnerability to AtCM (Figure 6).

### 5.1. Genetic Predisposition and Candidate SNPs

Susceptibility to DAtCM may be influenced by genetic predisposition, but current evidence directly linking specific SNPs to DAtCM remains indirect. *KCNJ11* encodes Kir6.2, a component of ATP‐sensitive potassium channels involved in insulin secretion, and *KCNJ11* rs5219 has been associated with T2DM susceptibility and T2DM‐related cardiovascular complications [70]. *HHEX* is related to pancreatic *β*‐cell function and insulin secretion, and *HHEX* rs1111875 has also been associated with T2DM susceptibility [70]. *SOD1* encodes Cu/Zn superoxide dismutase, an antioxidant enzyme involved in ROS detoxification; the *SOD1* + 35A/C variant has been associated with altered SOD activity and diabetic macrovascular complications [71, 72]. However, direct evidence linking these variants to DAtCM itself remains limited. Therefore, these variants should be viewed as candidate modifiers of metabolic and oxidative risk that may indirectly contribute to atrial vulnerability, rather than as established DAtCM risk loci. These candidate SNPs are summarized in Table 1.

**Table 1:** This table summarizes candidate SNPs with indirect relevance to DAtCM.

Gene	SNP locus	Function	Potential relevance to DAtCM	References
*KCNJ11*	rs5219	Regulates insulin secretion	Associated mainly with impaired insulin secretion and Type 2 diabetes susceptibility; it may indirectly influence DAtCM through metabolic risk, but direct DAtCM evidence is limited	[70]
*HHEX*	rs1111875	Regulates pancreatic development and *β*‐cell function	Associated mainly with *β*‐cell dysfunction and type 2 diabetes susceptibility; it may indirectly influence DAtCM through metabolic risk, but direct DAtCM evidence is limited	[70]
*SOD1*	rs2234694	Encodes copper–zinc superoxide dismutase	SOD1+35A/C (rs2234694) has been associated with altered SOD activity and diabetic macrovascular complications, but direct evidence for DAtCM is lacking; therefore, it is retained as a candidate oxidative stress modifier rather than an established DAtCM locus	[71]

### 5.2. Epigenetics

DAtCM involves a complex network of epigenetic regulatory mechanisms in its pathogenesis. Recent studies have indicated that the hyperglycemic environment influences dynamic modifications of DNA methylation, specific gene promoter methylation, posttranslational modifications of histones, and the expression of noncoding RNAs. These alterations subsequently drive metabolic dysregulation, oxidative stress, and fibrosis in cardiomyocytes [73].

DNA methylation is a critical epigenetic modification characterized by the formation of 5‐methylcytosine at specific cytosine residues in DNA [74]. This process is primarily catalyzed by DNA methyltransferases, which use S‐adenosylmethionine as the methyl donor [74]. In mammalian genomes, cytosine methylation occurs predominantly at CpG dinucleotides, and over 80% of cytosines in CpG contexts are methylated [75]. However, CpG island promoters are generally unmethylated in normal human cells [76]. Promoter CpG island methylation can repress gene transcription, mainly by limiting transcription factor binding and thereby altering chromatin accessibility [75]. Table 2 summarizes reported epigenetic alterations linked to diabetic cardiomyopathy or myocardial injury in diabetes; several methylation changes listed in this table are discussed in relation to oxidative stress, metabolic remodeling, cardiomyocyte dysfunction, and fibrosis.

**Table 2:** This table summarizes selected epigenetic alterations associated with diabetic cardiomyopathy or diabetes‐related myocardial injury.

Gene	Epigenetic alteration	Data sources	Relevance	Reference
*KEAP1*	Promoter hypomethylation	Myocardial specimens from T2DM patients with diabetic cardiomyopathy	Increases *KEAP1* expression, suppresses NRF2‐dependent antioxidant responses, and promotes oxidative stress	[77]
*NR1H3*	Promoter hypomethylation	STZ‐induced diabetic rat myocardium	Increases *NR1H3* mRNA and LXR*α* protein expression, contributing to lipid metabolic remodeling in diabetic myocardium	[78]
*JUND*	Promoter hypermethylation	STZ‐induced diabetic mice, *α*‐MHC‐JunD transgenic mice, and left ventricular specimens from T2DM patients	Reduces *JUND* mRNA levels and JunD protein expression, contributing to oxidative stress, inflammatory signaling, and myocardial dysfunction	[79]
*RASSF1A*	Promoter hypermethylation	DCM patients, STZ‐induced diabetic rats, and high glucose–treated cardiac fibroblasts	Reduces *RASSF1A* expression and promotes cardiac fibroblast proliferation and diabetic myocardial fibrosis	[80]
*HIF3A*	Intron 1 hypermethylation	Peripheral blood from T2DM patients with DCM	Reduces *HIF3A* expression and may disturb hypoxia‐related metabolic adaptation in diabetic cardiomyopathy	[81]
*GPX1*	Promoter hypermethylation	AGE‐exposed rats and primary neonatal SD rat cardiomyocytes	Reduces *GPX1* expression and antioxidant capacity, thereby enhancing ROS‐mediated cardiomyocyte apoptosis	[82]
*CDKN1A*	5 ^′^ flanking region methylation	Cardiac cells from diabetic patients and STZ‐induced diabetic rats	Increases p21 protein expression and disrupts cell cycle regulation during early molecular events in diabetic cardiomyopathy	[83]
*CCND1*	5 ^′^ flanking region methylation	Cardiac cells from diabetic patients and STZ‐induced diabetic rats	Reduces cyclin D1 protein expression and disrupts cell cycle regulation during early molecular events in diabetic cardiomyopathy	[83]

Epigenetic alterations are involved in multiple processes underlying diabetic myocardial injury, including oxidative stress, metabolic remodeling, cardiomyocyte dysfunction, and fibrosis. In the oxidative stress axis, hypomethylation of the *KEAP1* promoter can increase *KEAP1* transcription and KEAP1 protein expression. Because KEAP1 protein promotes the degradation of Nuclear Factor Erythroid 2–Related Factor 2 (NRF2), increased KEAP1 protein expression weakens NRF2‐dependent transcription of antioxidant enzymes, thereby reducing myocardial antioxidant capacity and aggravating redox imbalance under diabetic conditions [77]. For metabolic remodeling, hypomethylation of the *NR1H3* promoter can increase *NR1H3* mRNA and liver X receptor alpha (LXR*α*) protein expression in diabetic myocardium. Enhanced LXR*α* signaling is accompanied by upregulation of lipid‐handling genes involved in fatty acid uptake and cholesterol transport, including *ACSL3*, *CD36*, *ABCA1*, and *ABCG1*, thereby contributing to lipid metabolic remodeling in diabetic myocardium [78]. For cardiomyocyte dysfunction, promoter hypermethylation and other repressive epigenetic changes at the *JUND* locus can reduce *JUND* mRNA and JunD protein expression, thereby weakening antioxidant defense, enhancing oxidative stress and inflammatory signaling, and contributing to myocardial dysfunction [79]. In terms of fibrosis, promoter hypermethylation of *RASSF1A* can reduce *RASSF1A* expression, partly through Methyl‐CpG Binding Protein 2–mediated transcriptional repression in high‐glucose‐treated cardiac fibroblasts. Reduced *RASSF1A* expression removes an inhibitory brake on fibroblast proliferation, thereby promoting cardiac fibroblast expansion and diabetic myocardial fibrosis [80]. Other methylation changes involving *HIF3A*, *GPX1*, *CDKN1A*, and *CCND1* have also been linked to altered hypoxia adaptation, oxidative injury related to AGE accumulation, and early dysregulation of cell cycle control in diabetic cardiomyopathy or myocardial injury in diabetes [81–83].

### 5.3. Transcriptomics

Transcriptomic analysis has revealed broad diabetes‐related changes in cardiac gene expression, especially in pathways linked to AF, inflammation, and myocardial fibrosis [84, 85]. These findings support DAtCM biology, but many transcriptomic data derive from whole‐heart or ventricular‐enriched samples; atrial‐specific validation remains essential.

DM causes extensive transcriptomic changes in the gene expression of cardiac tissue. These changes mainly include metabolic pathway disorders, enhanced inflammatory responses, promoted apoptosis, and abnormal intercellular communication [84]. Furthermore, the upregulation of numerous metabolism‐associated genes in adipose tissue exacerbates the metabolic dysregulation in cardiac function [86].

In db/db mouse models of diabetes‐induced AF, MAPK10 expression is upregulated. MAPK10 promotes inflammation, fibrosis, electrical instability, and apoptosis in diabetic atria by engaging JNK signaling and activating NF‐*κ*B. It also increases the Bax/Bcl‐2 ratio, thereby contributing to cardiomyocyte apoptosis [85]. MAPK10 further enhances TGF‐*β* expression, which facilitates extracellular matrix production, collagen accumulation, and myocardial fibrosis. These structural changes can destabilize atrial electrophysiology and increase AF susceptibility (Figure 7) [84, 85].

Figure 7Downregulation of MAPK10 alleviates AF, structural remodeling, inflammation, fibrosis, and apoptosis induced by DM. (A) Representative atrial electrogram recordings of wild‐type (WT) and db/db mice injected with rAAV9‐sh‐GFP or rAAV9‐sh‐MAPK10. (B) Percentage of mice in which AF was induced by burst pacing (*n* = 15). (C) Average induced AF duration (*n* = 15). (D) Representative Masson′s trichrome staining for atrial fibrosis. (E) Quantification of fibrotic area (*n* = 6). (F) Representative M‐mode images of the left atrium at 12, 14, and 16 weeks. (G) Quantification of atrial width (*n* = 6). (H) Representative TUNEL staining of cardiomyocyte apoptosis in atrial sections. (I) Quantification of TUNEL‐positive nuclei (*n* = 6). Data are expressed as means ± SD. Differences were assessed by ANOVA with LSD testing.  ^∗∗∗^ *p* < 0.001, ∗∗*p* < 0.01, and  ^∗^ *p* < 0.05. Reproduced from Ref. [85] under the CC BY‐NC‐ND 4.0 license. Copyright 2022, the authors.

**Figure:**
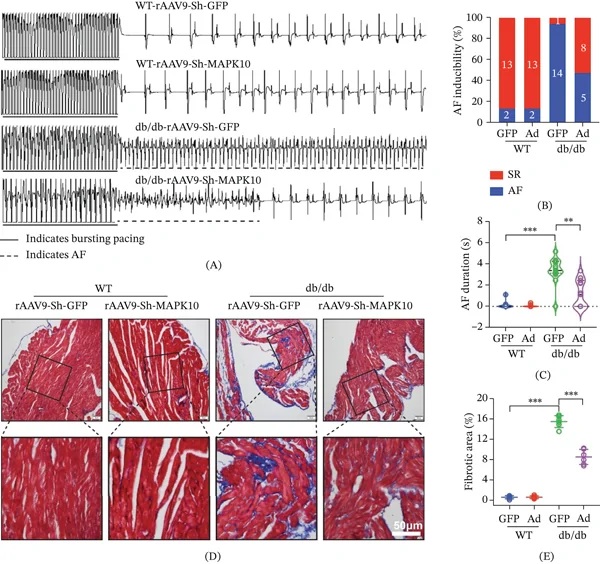

**Figure:**
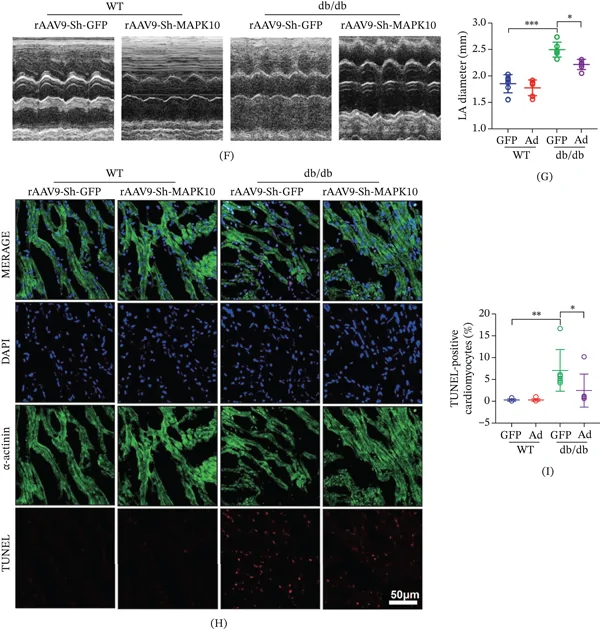

Under diabetic conditions, metabolism‐related genes such as Pdk4 and Angptl4 are upregulated in adipose‐associated cardiac tissue, increasing fatty acid oxidation and oxidative stress. These changes can injure cardiomyocytes and promote fibrosis. Elevated inflammatory mediators such as TNF‐*α* may further exacerbate cardiac inflammation through MAPK10 and NF‐*κ*B signaling [84, 86].

Single‐cell RNA sequencing indicates that diabetes alters cardiac cell–cell communication, especially interactions between fibroblasts and other myocardial cell populations. Enhanced intercellular signaling is associated with upregulation of fibrosis‐related genes, including Postn and TGF‐*β*, thereby promoting matrix remodeling and fibrotic progression [84].

The modification of the intercellular communication network encompasses the interactions among fibroblasts, endothelial cells, macrophages, and epicardial cells. Specifically, the expression of *Pdgfra* in fibroblasts is elevated, along with a significant increase in the expression of *Pdgfb* and *Pdgfd* in endothelial cells. These ligand–receptor interactions stimulate fibroblast proliferation and activation, thereby contributing to the progression of myocardial fibrosis [84].

**Figure 8:**
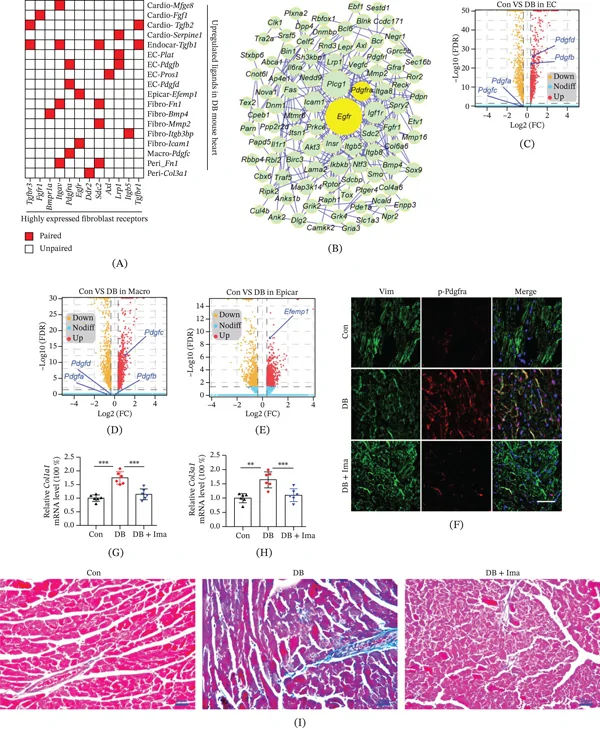
Identification of key ligand–receptor pairs associated with diabetic myocardial fibrosis in fibroblasts. (A) Heatmap showing highly expressed fibroblast receptors and upregulated ligands in each cell type in diabetic hearts. (B) Protein–protein interaction network of upregulated fibroblast genes. (C, D) Pdgfa, Pdgfb, Pdgfc, and Pdgfd expression in endothelial cells and macrophages. (E) Efemp1 expression in epicardial cells. (F) Representative immunofluorescence images of p‐Pdgfra in heart tissues from HFD/STZ‐treated mice with or without imatinib treatment (*n* = 6 mice per group; scale bar = 40 * μ*m). (G, H) Col1a1 and Col3a1 mRNA expression. (I) Representative Masson‐stained heart sections showing collagen deposition (*n* = 6 mice per group; scale bar = 20 * μ*m). Ima, imatinib mesylate; SEM, standard error of the mean; EC, endothelial cell; Macro, macrophage; Epicar, epicardial cell; Vim, vimentin. Reproduced from Ref. [84] under the CC BY 4.0 license. Copyright 2023, the authors.

In interactions between fibroblasts and macrophages, diabetic conditions increase *Pdgfc* expression in macrophages, and PDGFC may act on PDGFRA in fibroblasts, thereby enhancing fibroblast activation. In single‐cell ligand–receptor analysis, cardiac fibroblasts show high expression of receptor genes such as *Egfr* and *Pdgfra*, whereas their corresponding ligands may be supplied by other cardiac cell populations. Thus, PDGF ligands supplied by endothelial cells or macrophages may act on PDGFRA in fibroblasts, whereas EFEMP1 from epicardial cells may signal through EGFR in fibroblasts. Together, these interactions may contribute to diabetic myocardial fibrosis (Figure 8) [84].

Regarding communication between fibroblasts and epicardial cells, *Efemp1* is reported to be enriched in epicardial cells and may signal through EGFR in fibroblasts, augmenting fibrotic activation under diabetic conditions [84].

### 5.4. DM‐Associated Alterations in Gut Microbiota

In DAtCM, the gut–heart axis provides an upstream driver of atrial remodeling, inflammation, and lipotoxicity, making the microbiome a potential modifiable target for preventing or attenuating DAtCM.

The gut microbiota, as the largest symbiotic microbial community in the human body, plays a critical role in maintaining host glucose and lipid metabolic homeostasis through various mechanisms, including metabolic products, immune modulation, and barrier function. Dysbiosis of gut microbiota has been firmly linked to diabetes and its multiorgan complications. Specifically, in the context of diabetic cardiomyopathy, alterations in gut microbial composition—such as reduced butyrate‐producing bacteria and increased pathogenic bacteria—can mediate systemic inflammation, oxidative stress, and myocardial remodeling via the gut–heart axis. This process serves as a key link between metabolic disturbances and cardiac damage [87, 88]. This section will detail the characteristic changes in the gut microbiota associated with diabetes and focus on elucidating the molecular pathways by which these changes drive myocardial electrophysiological abnormalities, fibrosis, and functional decline.

Research into gut microbiota has revealed that, in individuals with DM, alterations in microbial composition are generally characterized by increased abundance of potentially harmful bacteria and reduced abundance of beneficial microorganisms. Alterations in the Firmicutes/Bacteroidetes ratio have also been reported, although the direction of this change varies among studies. This microbial imbalance may compromise intestinal barrier integrity, leading to increased gut permeability [88]. Consequently, bacteria and microbial products can translocate into the bloodstream, eliciting a systemic inflammatory response. Particularly in T2DM, gut microbial dysbiosis is associated with enrichment of potentially pathogenic bacteria, including *Escherichia coli* and some *Clostridium* species, together with a reduction in butyrate‐producing bacteria such as *Faecalibacterium prausnitzii* and *Eubacterium rectale*. This reduction in short‐chain fatty acid (SCFA)–producing bacteria may impair intestinal barrier integrity and immune regulation, while Gram‐negative bacteria–derived lipopolysaccharide (LPS) may further amplify systemic inflammation [88].

In addition to the damage resulting from the direct translocation of harmful bacterial metabolites into the bloodstream, the dysbiosis of the gut microbiota contributes to the progression of DAtCM by inducing an imbalance in microbial metabolic products [87, 88].

Trimethylamine N‐oxide (TMAO), generated from gut microbial metabolism of dietary precursors such as choline and carnitine, has been implicated in myocardial fibrosis and AF susceptibility. Current evidence mainly supports profibrotic and inflammatory mechanisms, including NLRP3 inflammasome activation, whereas direct evidence from DAtCM‐specific atrial models remains limited [87]. Reduced abundance of SCFA‐producing bacteria, including *Faecalibacterium prausnitzii* and *Eubacterium rectale*, may weaken gut‐barrier integrity, increase LPS translocation, and amplify systemic inflammation [88]. LPS can activate TLR4/NF‐*κ*B signaling and increase IL‐1*β* and TNF‐*α*, thereby contributing to myocardial inflammation and electrophysiological abnormalities [87, 88]. Diabetes‐associated alterations in gut bacteria involved in bile acid metabolism may disturb bile acid profiles and alter bile acid–farnesoid X receptor (FXR) signaling, a pathway involved in lipid metabolism and insulin sensitivity. However, direct evidence linking FXR‐related bile acid signaling to arrhythmogenesis remains limited, and this relationship should currently be interpreted as largely indirect and associative [88].

The microbiota–metabolism axis exerts a direct influence on myocardial pathology. Dysbiosis of the gut microbiota impacts the myocardium through multiple mechanisms, including systemic inflammation, oxidative stress, and autonomic nervous system remodeling. Proinflammatory factors such as LPS and TMAO activate myocardial macrophages to release ROS, resulting in mitochondrial dysfunction and cellular apoptosis [87, 88]. TMAO can also stimulate the atrial ganglion plexus, thereby enhancing sympathetic nerve activity and inducing atrial premature beats [87]. In addition, microbiota‐derived metabolites such as TMAO and indoxyl sulfate promote the proliferation of cardiac fibroblasts and collagen deposition by activating the TGF‐*β*/SMAD signaling pathway. Additionally, the activation of the NLRP3 inflammasome exacerbates myocardial interstitial fibrosis [87, 88]. These pathological alterations collectively result in myocardial electrophysiological abnormalities, structural remodeling, and functional decline, ultimately driving the progression of diabetic cardiomyopathy.

## 6. Management and Treatment Strategies

### 6.1. General Treatment

The management of DAtCM should address both metabolic regulation and cardiac protection. Its pathological foundation involves the interplay between metabolic disturbance, atrial remodeling, inflammation, microvascular injury, and thrombogenicity [89]. Current guidelines support multidisciplinary integrated management centered on lifestyle intervention, weight control, exercise rehabilitation, nutritional optimization, and cardiometabolic risk reduction [90, 91].

#### 6.1.1. Weight Management

For DAtCM, being overweight leads to insulin resistance, systemic inflammation, and cardiomyocyte fat accumulation, which accelerate atrial fibrosis and hypertrophy, so intentional weight management is a central upstream strategy to slow atrial remodeling.

Excess adiposity promotes insulin resistance, systemic inflammation, epicardial fat accumulation, atrial fibrosis, and LA structural remodeling [90]. The LEGACY study showed that sustained weight loss greater than 10% was associated with lower AF burden, improved arrhythmia‐free survival, and favorable reverse remodeling in an obese AF cohort [92]. However, LEGACY was not designed specifically for DAtCM, so these findings should be applied as supportive evidence for upstream risk factor modification rather than as proof of benefit specific to DAtCM. Gradual, sustained weight loss remains preferable to aggressive short‐term weight reduction because it helps preserve muscle mass and long‐term metabolic stability [92, 93].

Approaches encompass dietary management, exercise regimens, bariatric surgery, psychological counseling utilizing cognitive behavioral therapy to enhance dietary adherence, routine weight monitoring, and surveillance of metabolism‐associated biomarkers. For instance, gastric bypass surgery is recommended for individuals with a BMI of 35 or higher, accompanied by coexisting metabolic disorders. The long‐term outcomes of bariatric surgery surpass those of lifestyle interventions alone, especially in individuals with severe obesity. Regular body weight monitoring facilitates the achievement of weight loss and control objectives. Biomarker evaluation, including blood glucose, blood lipids, and other relevant indicators, enables the assessment of metabolic improvements and determination of intervention efficacy [90, 91].

In summary, achieving and sustaining a weight loss of over 10% through caloric restriction and aerobic exercise can significantly improve atrial remodeling and alleviate insulin resistance. Bariatric surgery is an appropriate intervention for individuals with severe obesity. Furthermore, dynamic monitoring in conjunction with multidisciplinary support plays a crucial role in maintaining long‐term health outcomes.

#### 6.1.2. Exercise Interventions

Because direct evidence for exercise intervention in DAtCM remains limited, exercise prescriptions should be individualized according to AF status, cardiopulmonary function, frailty, HF severity, and glycemic status. For patients with stable DAtCM, the aim is not to follow a fixed training formula, but to improve cardiorespiratory fitness, insulin sensitivity, weight control, and inflammatory burden in a safe manner. Exercise intensity can be guided by baseline assessment, such as cardiopulmonary exercise testing or a 6‐min walk test. Patients at high risk, including those with severe HF, exercise‐induced arrhythmia, or markedly reduced left ventricular ejection fraction, should begin exercise under supervised cardiac rehabilitation [90, 91].

Aerobic endurance activities, such as walking, cycling, or swimming, may be appropriate for clinically stable patients. Resistance training may be useful for obese patients or those with sarcopenia, whereas balance, flexibility, and mind–body exercises may benefit older or frail patients. Wearable monitoring may help guide activity progression, but exercise prescriptions should also consider symptoms, rhythm status, hypoglycemia risk, and comorbid vascular disease [90, 91].

Overall, exercise rehabilitation in DAtCM should emphasize individual risk stratification, gradual workload progression, multidimensional monitoring, and strategies to improve adherence, rather than fixed universal thresholds.

#### 6.1.3. Diet Modification

Cardioprotective dietary patterns are important in DAtCM patients because they reduce sugar intake, fat accumulation, and systemic inflammation. Adherence to cardioprotective dietary patterns, such as the Mediterranean and DASH diets, can significantly reduce the incidence of CVD. The PREDIMED trial demonstrated that adopting a Mediterranean diet rich in fruits, vegetables, whole grains, olive oil, and fish could reduce the risk of major adverse cardiovascular events (MACEs) by 25%–30% and lower the incidence of AF by nearly 20%. Similarly, the DASH diet has shown comparable benefits, contributing to approximately a 20% reduction in cardiovascular risk and producing reductions in blood pressure. These dietary improvements confer cardiovascular protection through multiple mechanisms, including attenuation of systemic inflammation, improvement in lipid metabolism, and enhancement of endothelial and myocardial function [90, 91].

#### 6.1.4. Smoking Cessation Interventions

In patients at risk for or with established DAtCM, smoking cessation is particularly important because smoking induces systemic inflammatory responses and oxidative stress, thereby accelerating atrial structural remodeling. Furthermore, smoking may compromise the efficacy of catheter ablation procedures for AF. Consequently, the implementation of smoking cessation interventions is essential. Such interventions can be categorized into three components: structured medical counseling, medication‐assisted smoking cessation therapy, and sustained multidisciplinary collaboration for long‐term intervention [90, 91].

Structured medical consultations were conducted in the outpatient clinic to provide individualized smoking cessation advice by clinicians [90, 91].

Among the medication‐assisted treatments, nicotine replacement therapy (e.g., nicotine patches and gum) is considered the first‐line option for individuals with moderate to severe nicotine dependence. If pharmacological interventions such as bupropion or varenicline are deemed necessary, the dosage should be adjusted based on the patient′s cardiovascular condition. Medication‐assisted therapy is recommended for smokers who demonstrate insufficient response to behavioral interventions alone [90, 91].

The multidisciplinary intervention is primarily aimed at enhancing long‐term adherence to smoking cessation, with the core objective being the improvement of sustained smoking abstinence rates over time. A specialized team comprising electrophysiologists, psychologists, and pharmacists was formed to provide systematic follow‐up care and psychological support [90, 91].

#### 6.1.5. Glycemic Management

Glycemic control encompasses individualized glycemic target setting, dietary modification, exercise intervention, weight management, and the use of antidiabetic medications. Notably, in selecting antidiabetic drugs, certain agents demonstrate additional benefits for cardiovascular‐related diseases beyond their effects on glycemic control. Therefore, some antidiabetic drugs may offer greater advantages in managing cardiovascular conditions compared to blood glucose control alone. This paragraph provides a brief overview of the impact of antidiabetic drugs on cardiovascular events, with more comprehensive details to be discussed in subsequent sections [89, 90].

SGLT2 inhibitors, sulfonylureas, thiazolidinediones, basal insulin, and rapid‐acting insulin are frequently used in clinical practice [89]. Thiazolidinediones may exacerbate fluid retention and HF risk, with rosiglitazone historically raising greater cardiovascular safety concerns and pioglitazone requiring caution in patients prone to HF [89, 94]. By contrast, SGLT2 inhibitors have shown cardiovascular and HF benefits in large outcome trials, including EMPA‐REG OUTCOME and DECLARE‐TIMI 58 in T2DM and the EMPEROR trials in HF populations [89, 95, 96]. These data support cardiometabolic benefits, but DAtCM‐specific atrial endpoints remain insufficiently studied.

#### 6.1.6. Blood Pressure Management

Given that atrial stretch and pressure overload lead to DAtCM progression, aggressive blood pressure control in patients with diabetes is essential to limit LA enlargement, alleviate wall stress, and reduce downstream AF risk. Chronic hypertension may result in myocardial fibrosis and microcirculatory dysfunction and expedite the onset of HF. Individuals with both diabetes and hypertension exhibit a two‐ to threefold increased risk of cardiovascular mortality. Furthermore, each 10‐mmHg reduction in systolic blood pressure is associated with a 20% decrease in the risk of major cardiovascular events. Effective blood pressure management can enhance myocardial energy metabolism, mitigate oxidative stress, and retard the structural alterations associated with diabetic cardiomyopathy [89, 90].

#### 6.1.7. Multidisciplinary Integrated Management

The pathological basis of DAtCM involves a complex interaction between metabolic dysregulation, atrial remodeling, inflammation, and microvascular injury [89]. A single‐discipline model is unlikely to address the full range of required interventions, including metabolic optimization, cardiac rehabilitation, nutrition, sleep medicine, and rhythm surveillance. Patients with both diabetes and AF who receive only cardiovascular treatment remain at markedly elevated long‐term cardiovascular risk compared with the general population [90]. Multidisciplinary care can integrate endocrinology, cardiology, nutrition, rehabilitation, sleep medicine, psychology, and pharmacy to support dynamic risk stratification, AF burden monitoring, and sustained adherence [89, 90]. Such collaboration is, therefore, central to interrupting the metabolic–cardiovascular cycle that drives DAtCM progression.

### 6.2. Medicine Treatment

#### 6.2.1. Anticoagulant Therapy

In DAtCM, anticoagulation therapy does not address the atrial pathological changes; however, it plays a critical role in reducing the risk of thromboembolism associated with the condition.

In patients with AF and DM, oral anticoagulant selection should follow current AF guidelines and individualized assessment of stroke risk, bleeding risk, renal function, drug interactions, and patient preference. Rivaroxaban plus aspirin reduced cardiovascular events in stable atherosclerotic disease in the COMPASS trial, including in patients with diabetes, but this regimen is not a general substitute for AF anticoagulation and should be used only in appropriate vascular indications [97]. Dabigatran and warfarin have broadly comparable relative efficacy and safety patterns in patients with and without diabetes, and diabetic status alone does not mandate dosage adjustment [98].

For patients with AF and DM who also have atrial myocardial disease, anticoagulation should be based on comprehensive stroke risk stratification, such as the CHA2DS2‐VASc score and current AF guidelines. DM alone is not an independent indication for anticoagulation in the absence of AF or another guideline‐supported indication [99]. For patients with atrial cardiopathy but no documented AF, the ARCADIA trial showed that apixaban did not reduce recurrent stroke compared with aspirin in patients with recent cryptogenic ischemic stroke and prespecified atrial cardiopathy biomarkers [100]. This result should not be generalized to all DAtCM patients, but it supports a cautious approach: Optimize risk factors, monitor rhythm, and initiate anticoagulation when AF or another guideline‐supported indication is present [100, 101].

#### 6.2.2. Ventricular Rate Treatment

In the MERIT‐HF study, the treatment effect was not found to be different for diabetic and nondiabetic patients treated with metoprolol [102]. CIBIS II shows that bisoprolol has a similar therapeutic effect in patients with and without diabetes [103]. In both these studies, the starting dose and dose escalation were the same in patients with diabetes as in those without diabetes. Metoprolol is more effective in diabetic patients with CHF. Bisoprolol has a wider applicability in diabetic patients [102, 103].

#### 6.2.3. Rhythm Treatment

The ATHENA study demonstrated that dronedarone was equally effective in reducing rates of cardiovascular hospitalization and mortality in both diabetic and nondiabetic patients. However, findings from the EURIDIS/ADONIS study indicated that the reduction in AF recurrence among diabetic patients did not achieve statistical significance, whereas a statistically significant 25% reduction in AF recurrence was observed in nondiabetic patients. Notably, dronedarone may offer potential benefits in heart rate control among patients with diabetes. During the first AF episode, the mean heart rate in diabetic patients receiving dronedarone decreased by 16.5 beats per minute, compared to a reduction of 8 beats per minute in both the placebo group and nondiabetic patients. Regarding the drug dosage, the same dosage and treatment regimen for dronedarone were administered across all patient groups, irrespective of diabetes status [104].

#### 6.2.4. Addressing Upstream Factors

Although most outcome trials of cardiometabolic drugs have focused on ventricular function, HF, or AF, several classes of agents target pathways that are central to DAtCM pathophysiology, including atrial structural remodeling, oxidative stress, and systemic inflammation. In this section, we summarize how these therapies might be repurposed or prioritized as upstream interventions in patients with or at risk for DAtCM.

##### 6.2.4.1. SGLT2 Inhibitors

Empagliflozin improved cardiovascular outcomes in EMPA‐REG OUTCOME, a trial of patients with T2DM and established CVD. Empagliflozin reduced cardiovascular death by 38%, hospitalization for HF by 35%, and three‐point MACE by 14% relative to placebo [95]. These results support cardiovascular protection in high‐risk diabetes, but they do not prove a DAtCM‐specific therapeutic effect. Empagliflozin should, therefore, be discussed as a cardiometabolic intervention with plausible upstream relevance to DAtCM rather than as a validated atrial remodeling therapy.

**Figure 9:**
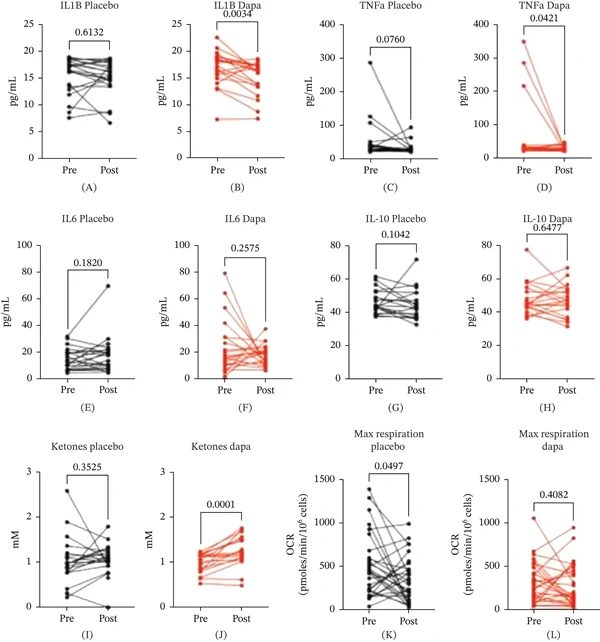
Levels before and after 12 months of treatment with placebo or dapagliflozin. (A–H) Plasma IL‐1*β*, TNF‐*α*, IL‐6, and IL‐10. (I, J) Plasma ketones (acetoacetate + *β*‐hydroxybutyrate). (K, L) Peripheral blood mononuclear cell maximal oxygen consumption rate (OCR). Exact *p* values are shown above each paired comparison. *p* values were calculated using a paired two‐tailed *t*‐test when normality assumptions were met; otherwise, a nonparametric paired test was used. For plasma cytokines and ketones, statistical significance was set at *p* ≤ 0.0083 after Bonferroni correction. Nominal *p* values that did not meet this threshold were shown for reference but were not considered statistically significant after correction. Reproduced from Ref. [105] under the CC BY 4.0 license. Copyright 2024, the authors.

Dapagliflozin may attenuate systemic inflammation and metabolic dysfunction. In a randomized placebo‐controlled study of patients with T2DM without known HF, 1 year of dapagliflozin was associated with a statistically significant within‐group reduction in plasma IL‐1*β* from an approximate baseline level of 17–18 to 15–16 pg/mL, corresponding to an absolute decrease of 1.8 pg/mL [105]. However, the absolute reduction was small, and the study did not show significant improvement in CMRI measures, including myocardial strain, extracellular volume fraction, or T2 relaxation time. Therefore, this IL‐1*β* change should be interpreted cautiously as biomarker‐level evidence of systemic anti‐inflammatory activity, rather than as proof of clinically meaningful benefit or reversal of atrial remodeling in DAtCM (Figure 9).

**Figure 10:**
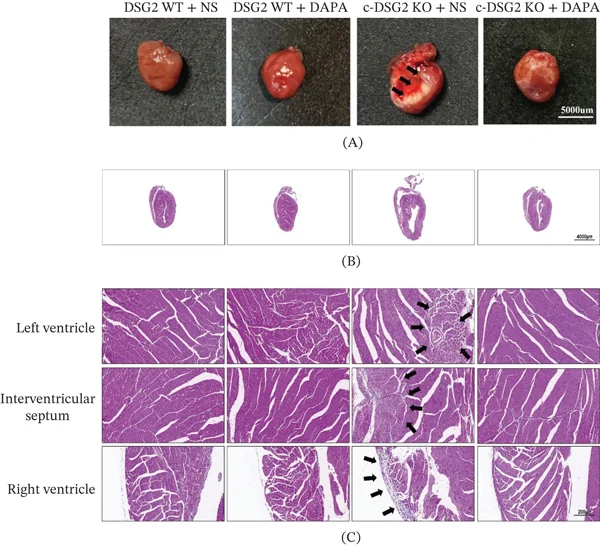
Dapagliflozin attenuated cardiac fibrofatty replacement in arrhythmogenic cardiomyopathy mice. (A) Gross heart appearance (scale bar = 5000 * μ*m). (B) Representative microscopic appearance among groups (scale bar = 4000 * μ*m). (C) Representative hematoxylin and eosin staining of left ventricle, interventricular septum, and right ventricle sections (scale bar = 200 * μ*m). DAPA, dapagliflozin; c‐DSG2 KO, cardiomyocyte‐specific Dsg2 Exon‐11 knockout; WT, wild type; NS, normal saline. Reproduced from Ref. [106] under the CC BY license. Copyright 2022, the authors.

Dapagliflozin can also increase ketone body availability, improve glycemic control, and support weight reduction. In the same study, total plasma ketone bodies, including *β*‐hydroxybutyrate and acetoacetate, increased by 0.26 mM in the dapagliflozin‐treated group [105]. These metabolic shifts may improve myocardial energy efficiency, but direct evidence for slowing atrial lipid accumulation in DAtCM is lacking. In an arrhythmogenic cardiomyopathy mouse model, dapagliflozin reduced cardiac fibrofatty replacement, suggesting broader antiremodeling potential that requires validation in diabetic atrial models [106] (Figure 10).

##### 6.2.4.2. Dual SGLT2 and SGLT1 Inhibitors

In addition to its effect of lowering blood sugar, the SOLOIST‐WHF trial revealed that the MACE of the sotagliflozin group was reduced by 34%. The incidence of first and subsequent hospitalizations for HF was 51.3% in the sotagliflozin group compared to 77% in the placebo group. Overall, sotagliflozin significantly reduced the incidence of MACE, with no serious adverse reactions or side effects reported [107].

##### 6.2.4.3. Mineralocorticoid Receptor Antagonist (MRA)

Spironolactone may delay diabetic myocardial remodeling by improving diastolic function and inhibiting fibrosis [108, 109]. In the trial by Jellis et al., 6 months of spironolactone at 25 mg daily was associated with changes in Doppler and tissue characterization indices, including reduced integrated backscatter, suggesting less myocardial fibrosis [108]. The HOMAGE trial also showed that spironolactone lowered Type I procollagen carboxy‐terminal peptide and NT‐proBNP in patients at risk of HF, including patients with diabetes, supporting a potential antifibrotic effect [109].

Finerenone is a nonsteroidal MRA that may delay diabetic myocardial injury through antifibrotic and anti‐inflammatory effects. In the FIDELITY pooled analysis, finerenone reduced the composite cardiovascular outcome, defined as cardiovascular death, nonfatal myocardial infarction, nonfatal stroke, or hospitalization for HF, by 14% as a relative risk reduction in patients with T2DM and chronic kidney disease. The modest systolic blood pressure reduction of approximately 2.6–3.2 mmHg should be viewed as a secondary hemodynamic effect rather than the primary efficacy argument [110]. FIGARO‐DKD further showed reductions in HF hospitalization and cardiovascular outcomes, supporting finerenone as a cardiometabolic and renal‐protective therapy with plausible relevance to DAtCM [111].

Given the capacity of MRA to mitigate myocardial fibrosis, it may serve as adjunctive therapy to improve the prognosis of patients with DAtCM [112].

##### 6.2.4.4. Trimetazidine (TMZ)

TMZ may improve myocardial energy metabolism in selected diabetic patients, but evidence for DAtCM is limited and indirect [113, 114]. It should be considered an adjunctive therapy with limited evidence rather than a core disease‐modifying treatment.

In terms of diastolic function, the study conducted by Serag et al. revealed LA volume index in the TMZ group decreased from 31.54 to 28.77 mL/m^2^, representing a reduction of 6.99%, and the mean *e* ^′^ velocity increased from 7.7 to 8.16 cm/s, corresponding to an increase of 8.46% [114]. In the study conducted by Jatain et al., the *E*/*A* ratio in the TMZ group decreased from 1.94 to 1.19, while the myocardial performance index improved from 0.8 to 0.7, indicating a notable enhancement in myocardial function [113]. Among the biomarkers observed, the BNP level decreased by 66.8%, from 744.7 to 248.3 pg/mL [113]. The LDL‐C level decreased from 119.29 to 103.43 mg/dL [114].

##### 6.2.4.5. Angiotensin Receptor–Neprilysin Inhibitors (ARNIs)

ARNIs may protect diabetic myocardium by attenuating cardiac remodeling, fibrosis, apoptosis, oxidative stress, and inflammatory signaling [115]. However, current evidence mainly comes from HF populations and experimental diabetic cardiomyopathy models. Therefore, ARNIs may be considered when guideline‐supported indications such as HF are present, but randomized controlled trials with DAtCM‐specific atrial endpoints are needed before ARNI therapy can be recommended specifically for DAtCM.

##### 6.2.4.6. Potential Target NR4A3

NR4A3 has emerged as a promising experimental target for diabetes‐induced atrial remodeling. In db/db mouse models, NR4A3 upregulation attenuated atrial hypertrophy, fibrosis, oxidative stress, and mitochondrial dysfunction [116]. These data support further mechanistic study, but they do not yet establish NR4A3 as a clinically validated therapeutic target for DAtCM.

Cardiac‐specific NR4A3 overexpression suppressed atrial structural remodeling in db/db mice. NR4A3 upregulation reduced the LA weight‐to‐body‐weight ratio, LA area, interstitial fibrosis, and cardiomyocyte cross‐sectional area. ANP and Fibrillar Collagens I and III were also downregulated, aligning molecular and histological readouts [116]. These findings suggest that the beneficial effects of NR4A3 overexpression were observed mainly under diabetic conditions, as nondiabetic control mice showed no measurable changes in these endpoints. However, this should be interpreted as preclinical evidence rather than proof of clinical specificity (Figure 11).

Figure 11Cardiac‐specific overexpression of NR4A3 attenuates diabetes‐induced atrial remodeling in db/db mice. (A) Representative whole‐heart images from db/m and db/db mice 16 weeks after injection with AAV9‐cTNT‐Nr4a3 or AAV9‐cTNT‐Ctrl (scale bar = 1 mm). (B) Representative long‐axis images from two‐dimensional echocardiography. (C) Representative Masson staining (scale bar = 50 * μ*m). (D) Representative WGA staining (scale bar = 20 * μ*m). (E) Left atrial weight (LAW)‐to‐total body weight (TBW) ratio (*n* = 8 per group). (F) Left atrial area (*n* = 8 per group). (G) Quantification of fibrosis area (*n* = 8 per group). (H) Quantification of cardiomyocyte area (*n* = 8 per group). (I) Immunoblotting and (J) quantitation of ANP protein level (*n* = 6 per group). (K) Immunoblotting and (L, M) quantitation of COL1A1 and COL3A1 protein levels (*n* = 6 per group). All data are presented as the mean ± SD. For statistical analysis, one‐way ANOVA with Bonferroni post hoc analysis was used.  ^∗^ *p* < 0.05,  ^∗∗^ *p* < 0.01,  ^∗∗∗^ *p* < 0.001, and  ^∗∗∗∗^ *p* < 0.0001. Reproduced from Ref. [116] under the CC BY‐NC‐ND 4.0 license. Copyright 2024, the authors.

**Figure:**
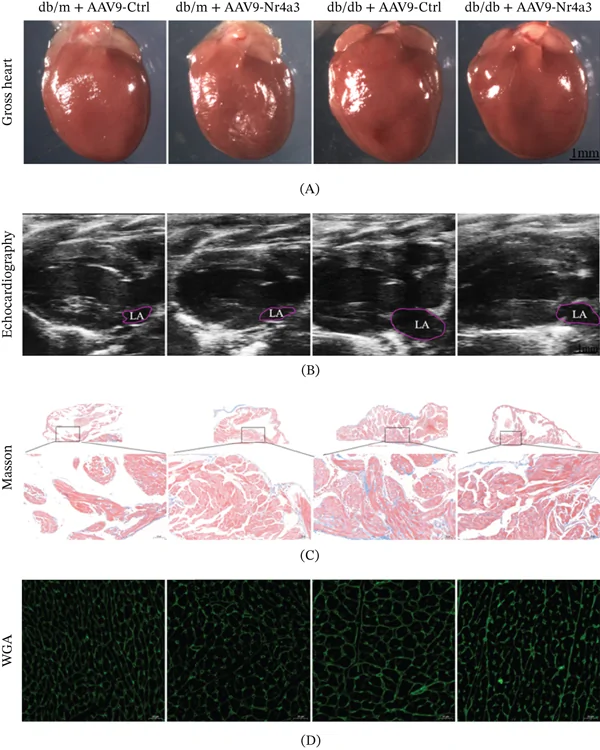

**Figure:**
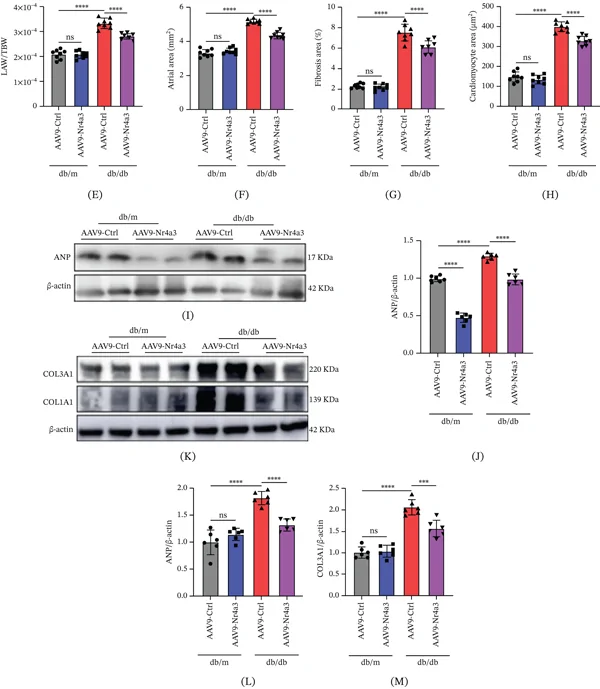

Electrophysiologically, NR4A3 upregulation reduced pacing‐induced AF vulnerability in db/db mice. At the mitochondrial level, NR4A3 maintained Succinate Dehydrogenase Complex Flavoprotein Subunit A expression, improved Complex II–linked respiration, stabilized mitochondrial membrane potential, and reduced ROS production in atrial tissue and HL‐1 atrial cardiomyocytes [116]. However, clinical translation remains challenging. As NR4A3 is an orphan nuclear receptor with transcriptional regulatory activity, therapeutic modulation of this target requires further clarification of small‐molecule druggability, target selectivity, potential off‐target transcriptional effects, and long‐term safety [117]. In addition, cardiac or atrial tissue–specific delivery remains an important practical challenge for translating NR4A3 modulation into DAtCM therapy. Additional animal models, human atrial tissue validation, and pharmacologic development are required before NR4A3 can be considered a clinically actionable target for DAtCM.

### 6.3. Operative Treatment

#### 6.3.1. Left Atrial Appendage Occlusion (LAAO)

Long‐term outcomes of LAAO, including occlusion success, thromboembolic events, mortality, and bleeding complications, have shown no statistically significant differences between patients with DM and those without DM in available observational data [118]. These findings suggest that DM alone does not necessarily reduce LAAO efficacy, although procedural selection should still follow standard indications and individualized risk assessment.

#### 6.3.2. Catheter Ablation

Catheter ablation outcomes differ between patients with and without DM. Wang et al. reported an AF recurrence rate of 56.9% in the DM group compared with 33.9% in the non‐DM group [119]. Studies by Lin et al. and Wang et al. similarly showed lower AF‐free survival among patients with DM [119, 120]. Symptom improvement, assessed with the Mayo AF Symptom Inventory, occurred in both diabetic and nondiabetic groups without a significant between‐group difference, indicating that catheter ablation can still improve quality of life despite higher recurrence risk in DM [119]. Procedural indications and rhythm management strategies should, therefore, follow standard AF guidance while placing greater emphasis on upstream risk factor control in patients with diabetes [120].

Glycemic control is an important modifiable factor after AF ablation in patients with diabetes. A 1% reduction in HbA1c during the 12 months before ablation has been associated with an approximately 28.6% lower risk of AF recurrence [121]. Optimizing glycemic status may, therefore, improve ablation prognosis in patients with DAtCM and concomitant AF, although prospective trials with atrial remodeling endpoints are still needed.

## 7. The Application of AI in Diagnosis and Prediction of Risk Endpoints

In recent years, AI has become a promising tool for diagnosis and risk prediction in diabetes and CVD. In diabetes, machine learning–based clustering models can integrate echocardiographic parameters and cardiac biomarkers to identify high‐risk diabetic cardiomyopathy phenotypes, but DAtCM‐specific unsupervised clustering models remain limited [122]. Supervised learning approaches, including convolutional neural networks (CNNs), have been used for cardiomyopathy phenotyping from ECG, echocardiography, and CMR data [122–124]. AI‐enhanced ECG (AI‐ECG) can detect subtle signatures associated with left ventricular dysfunction, AF risk, HCM, and atrial myopathy [123, 125]. Wearable ECG and photoplethysmography (PPG) may expand rhythm monitoring, but performance should be validated in diabetic populations with different comorbidity burdens. Overall, AI provides a useful framework for DAtCM research, but clinical implementation requires prospective, externally validated, and explainable models.

### 7.1. The Application of AI in the Diagnosis and Assessment of AtCM

Because DAtCM is conceptualized as an AtCM phenotype arising in the setting of DM, AI approaches developed to detect and quantify AtCM may be adapted to identify diabetes‐specific atrial patterns.

Cardiomyopathy phenotypes such as dilated cardiomyopathy, HCM, and cardiac amyloidosis differ in imaging and biomarker profiles. AI‐based echocardiographic analysis has shown potential for automated phenotyping in established cardiomyopathies [124]. For example, Hwang et al. developed a CNN‐LSTM model based on transthoracic echocardiography and reported high classification performance for cardiac amyloidosis, HCM, and hypertensive heart disease [126]. This study is relevant as proof of concept for echocardiography‐based phenotyping, but these diseases are not direct DAtCM differential diagnoses, and the results should not be interpreted as DAtCM validation.

For LA myopathy, a CNN‐based AI‐enabled 12‐lead ECG algorithm generated an AI‐ECG probability score associated with LA remodeling, LA functional impairment, and future AF risk. Among 306 patients without prior AF, each 10% increase in the AI‐ECG score was associated with a 31% higher risk of new‐onset AF [125]. This suggests that AI‐ECG may detect an atrial myopathic substrate before overt AF. However, because the original algorithm was developed using sinus rhythm ECGs and the new‐onset AF analysis was restricted to patients without prior AF, the applicability of this AI‐ECG approach in DAtCM patients with established AF remains uncertain.

The quantitative assessment of cardiac structural abnormalities combines CMR and deep learning to automatically analyze parameters such as left ventricular ejection fraction and left ventricular mass. In addition, deep learning has been applied to myocardial tissue characterization and scar quantification on late gadolinium enhancement CMR to assess myocardial fibrosis [127]. It has been demonstrated that, while achieving precision comparable to manual CMR analysis, AI‐based CMR analysis is 186 times faster than human analysis [128].

### 7.2. The Application of AI in Identifying High‐Risk Phenotypes of Diabetic Cardiomyopathy

Although the AI models used for diabetic cardiomyopathy focus on the overall cardiomyopathy phenotype, the same framework can be extended to identify the phenotype dominated by atrial lesions.

In diabetic cardiomyopathy, AI has been applied to identify phenotypic subgroups and predict high‐risk diabetic cardiomyopathy. One study used multiple machine learning methods, including deep neural network (DNN), decision tree, random forest, and logistic regression models, to classify high‐risk diabetic cardiomyopathy phenotypes [122]. The OptiML platform was used for Bayesian hyperparameter optimization and feature importance assessment. The DNN model performed best in internal validation, with an AUC of 0.96, while random forest and logistic regression also performed well, with AUC values of 0.94 and 0.937, respectively [122]. These results support machine learning for diabetic cardiomyopathy phenotyping, but atrial‐specific DAtCM validation is still needed.

### 7.3. AI‐ECG in Identifying CAN in DM

CAN may contribute to structural remodeling and electrophysiological vulnerability relevant to DAtCM progression, making CAN screening clinically relevant [129]. In the study by Irlik et al., an AI‐ECG model was developed using standard 12‐lead ECG recordings of 10‐s duration and a support vector machine (SVM) combined with feature extraction methods, including long short‐term memory–based feature extraction and motif/discord analysis [130]. Compared with the previously reported AUC of 0.73 for standard deviation of normal‐to‐normal intervals–based CAN detection [131], the SVM model combining motif and discord features achieved an AUC of 0.93 for definite/severe CAN classification [130].

### 7.4. The Application of AI in Risk Prediction and Monitoring of AF

Because AF is a major clinical endpoint of DAtCM, risk prediction and longitudinal rhythm monitoring are central to patient stratification.

ECG data can be utilized by AI to predict the risk of AF and LA abnormalities. In a study employing AI analysis of ECG data, patients were stratified into four groups based on their predicted AF risk probability: 0%–13%, 14%–42%, 43%–68%, and 69%–100%. The results indicated that patients in the highest risk group exhibited significantly larger LA volumes (left atrium reservoir strain decreased from 40.8 ± 18.1 to 15.7 ± 7.1, *p* < 0.001), reduced atrial compliance (booster strain decreased from 18.6 ± 10.1 to 10.5 ± 7.4, *p* = 0.040), and more severe mitral and tricuspid regurgitation (the proportion of moderate‐to‐severe mitral regurgitation increased from 12% to 28%, *p* = 0.002). Furthermore, validation of the AI prediction model using clinically diagnosed AF patients within 31 days yielded an AUC of 0.87, underscoring its significant value in predicting AF [125].

Wearable devices that use PPG‐based sensors have expanded AF screening and monitoring. In the Apple Heart Study, irregular pulse notifications had a positive predictive value of 84% for AF during simultaneous ECG patch monitoring [132]. The HUAWEI Heart Study reported a positive predictive value of 91.6% for PPG‐based AF detection [133]. These studies were conducted in broad screening populations rather than diabetic or DAtCM‐specific cohorts. Therefore, diagnostic performance, including false‐positive and false‐negative rates, may differ in patients with diabetes, autonomic neuropathy, vascular disease, or established atrial disease.

Machine learning–based analysis of atrial fibrosis distribution derived from late gadolinium enhancement magnetic resonance imaging, combined with personalized mechanistic simulations, has been applied to predict AF recurrence following pulmonary vein isolation. The predictive model demonstrated an average validation sensitivity of 82%, specificity of 89%, and AUC of 0.82 [134].

### 7.5. AI‐Assisted Prediction of AF Recurrence After Catheter Ablation in Diabetics

Because DM is associated with an increased risk of AF recurrence after catheter ablation, machine learning models may help identify patients who need intensified upstream management and rhythm surveillance. In a recent study of 430 patients undergoing first‐time radiofrequency catheter ablation for nonvalvular AF, a light gradient boosting machine (LightGBM) model achieved an accuracy of 0.721 and an AUC of 0.848 for recurrence prediction [135]. This performance suggests potential clinical relevance but remains moderate, and the cohort was not specifically designed for patients with diabetes. Therefore, the model should be viewed as a candidate risk stratification approach requiring DAtCM‐focused validation before clinical use.

## 8. Future Research Directions and Challenges

DAtCM is increasingly recognized as a distinct and clinically significant condition, yet much of the current evidence still derives from ventricular cardiomyopathy, HF, and AF research paradigms [5]. Future studies should prioritize atrial‐specific mechanisms, measurements, and clinical endpoints. Although consensus frameworks such as the EHRAS classification provide an important foundation [6], phenotypic heterogeneity, multimorbidity, and stage‐specific disease trajectories remain insufficiently addressed. Dedicated DAtCM cohorts and trial designs are, therefore, required.

Mechanistically, candidate therapeutic nodes require testing in translational atrial models. Calcium‐handling abnormalities, including increased RyR2 phosphorylation and CaMKII activation [41], and sodium/potassium channel remodeling, including NF‐*κ*B‐mediated SCN5A transcriptional repression [44] and TASK‐1 acid sensitivity [46], may contribute to electrophysiological vulnerability. Fibrotic and inflammatory pathways, including ET‐1‐mediated EndMT [66], PARP‐1/IKK*α*/NF‐*κ*B signaling, and NLRP3 inflammasome activation, represent plausible targets for limiting atrial remodeling [48, 87]. Emerging molecular pathways and regulatory mechanisms, including Ang II‐P2X7R‐HuR‐regulated ferroptosis [136], KEAP1‐NRF2‐mediated oxidative stress [77], and microRNA regulation such as miR‐133a [51], may expand the therapeutic landscape. The gut–heart axis, involving TMAO, SCFAs, and bile acid–FXR signaling, also warrants investigation as a potential microbiome–metabolic intervention pathway [87, 88]. These targets should be evaluated as disease‐modifying strategies and as upstream modifiers that may improve the durability of rhythm control interventions.

A critical limitation of current evidence is that therapeutic effects for many medicines, including SGLT2 inhibitors, ARNIs, and TMZ, are largely extrapolated from global cardiovascular/HF populations, diabetic complication evidence, or ventricular‐focused diabetic cardiomyopathy cohorts, with DAtCM‐specific mechanisms and causal links to atrial remodeling inferred but not directly proven [95, 114, 115]. These therapies require randomized studies or prospective mechanistic cohorts with atrial endpoints before they can be considered DAtCM‐specific interventions. In addition to evaluating existing medicines, there is a need to translate experimentally validated targets into clinical applications. NR4A3 upregulation has shown beneficial effects on atrial remodeling and mitochondrial dysfunction in diabetic mouse models, but it remains preclinical [116].

### 8.1. AI‐Based Early Detection of DAtCM

One concrete translational direction is to establish and prospectively validate an early screening and risk stratification pathway for DAtCM based on AI‐ECG, imaging, and biomarkers. AI‐ECG algorithms and wearable PPG/ECG devices can detect subtle patterns of atrial remodeling and predict AF and HF events that are difficult to recognize clinically, whereas natriuretic peptides and high‐sensitivity cardiac troponins may refine biological risk assessment [122, 127].

In a first step, a multicenter cohort of patients with Type 1 and Type 2 diabetes without known AF would undergo standardized sinus rhythm 12‐lead ECG recording and echocardiography, including LA volume and strain. Contrast‐enhanced CMR could be used in selected research sites to quantify atrial fibrosis, but routine deployment may be limited by cost, scanner availability, contraindications, and standardization of atrial fibrosis segmentation. In a second step, the AI score would be integrated with circulating biomarkers, including NT‐proBNP, hs‐cTnT, hs‐cTnI, and fibrosis markers, and with genetic or epigenetic candidates implicated in diabetic cardiovascular complications, using supervised learning methods such as DNN [70, 72, 122, 137]. Independent validation should assess discrimination, calibration, and clinical utility for incident AF, HF hospitalization, imaging‐defined progression of atrial remodeling or fibrosis, and thromboembolic events. Cost‐effectiveness, workflow burden, equity of access, and interpretability should be evaluated before routine clinical implementation.

### 8.2. Staged Translational Pathway for NR4A3 Modulation in DAtCM

Another translational direction is to develop a staged preclinical and early clinical framework for NR4A3 modulation in DAtCM. Preclinical studies in db/db mice show that cardiac‐specific NR4A3 overexpression attenuates atrial hypertrophy and fibrosis, improves mitochondrial respiration, reduces oxidative stress, and lowers AF vulnerability [116]. Existing NR4A family drug development provides early evidence of pharmacological tractability but also underscores the preliminary stage of this field. For NR4A3, also known as NOR‐1, only a limited number of direct modulators have been reported; fragment screening identified several NOR‐1 ligand chemotypes, including inverse agonists with low‐micromolar potency, indicating that direct pharmacological modulation is possible, although therapeutic applicability remains unproven [138]. More recent screening of fatty acid mimetics identified multiple NR4A agonist and inverse agonist scaffolds, and optimized compounds showed potent agonist activity across NR4A receptors, including NOR‐1, although off‐target profiles, cardiovascular validation, and disease‐context efficacy remain unresolved [139]. Before human trials, however, NR4A3‐targeted therapy requires evidence that the target is pharmacologically tractable in the relevant direction and tissue context. Early work should define whether small molecules, RNA‐based approaches, or gene delivery strategies can modulate NR4A3 with adequate potency, specificity, atrial delivery, and safety. Candidate interventions should be tested across complementary DAtCM models using harmonized endpoints, including LA volume and strain, histological fibrosis burden, mitochondrial function, and inducible AF. Only after these requirements are met would a Phase Ib/IIa trial incorporating atrial imaging and biomarker endpoints be justified [117].

If such a trial becomes feasible, primary endpoints should focus on safety, target engagement, and changes in atrial strain or fibrosis over 6–12 months. Secondary endpoints could include AF burden, HF events, and patient‐reported outcomes. AI models integrating AI‐ECG features, imaging results, and multiomics signatures could then help identify DAtCM phenotypes most likely to respond to NR4A3 modulation and refine enrichment strategies for later platform or Phase II/III trials.

## 9. Conclusions

In summary, DAtCM is an increasingly important complication of diabetes, but its mechanisms and management remain less mature than those of ventricular diabetic cardiomyopathy. Diabetes promotes atrial fibrosis, altered calcium and sodium handling, metabolic stress, inflammation, microvascular dysfunction, autonomic imbalance, and thrombogenicity. These processes impair atrial function and increase susceptibility to AF, HF, and embolic events. Multiomics studies and AI‐enabled phenotyping may improve early detection and risk stratification, but most evidence remains indirect and experimental or derived from non‐DAtCM cohorts. Future progress will require atrial‐specific cohorts, standardized imaging and biomarker endpoints, validated AI models, and trials that test whether upstream cardiometabolic or targeted interventions can slow DAtCM progression.

**Nomenclature**
AF: atrial fibrillation
AGEs: advanced glycation end products
AHA: American Heart Association
AI: artificial intelligence
AMPK: adenosine monophosphate–activated protein kinase
Ang II: Angiotensin II
ANP: atrial natriuretic peptide
APHRS: Asian Pacific Heart Rhythm Society
ARNI: angiotensin receptor–neprilysin inhibitor
AtCM: atrial cardiomyopathy
AUC: area under the curve
BMI: body mass index
CaMKII: Calcium/Calmodulin‐Dependent Kinase II
CAN: cardiac autonomic neuropathy
CMRI: cardiac magnetic resonance imaging
CNN: convolutional neural network
CTGF: connective tissue growth factor
CVD: cardiovascular disease
DADs: delayed afterdepolarizations
DAtCM: diabetic atrial cardiomyopathy
DM: diabetes mellitus
DNN: deep neural network
EADs: early afterdepolarizations
ECG: electrocardiogram
EHRA: European Heart Rhythm Association
EndMT: endothelial‐to‐mesenchymal transition
ET‐1: Endothelin‐1
FFAs: free fatty acids
FXR: farnesoid X receptor
HCM: hypertrophic cardiomyopathy
HF: heart failure
HRS: Heart Rhythm Society
hs‐cTnI: high‐sensitivity cardiac troponin I
hs‐cTnT: high‐sensitivity cardiac troponin T
IKK*α*
: I*κ*B kinase *α*
IL: interleukin
LA: left atrium
LAAO: left atrial appendage occlusion
LAHRS: Latin American Heart Rhythm Society
LPS: lipopolysaccharide
MACE: major adverse cardiovascular event
MAPK: mitogen‐activated protein kinase
METs: metabolic equivalents
MMPs: matrix metalloproteinases
MRA: mineralocorticoid receptor antagonist
NCX: sodium–calcium exchanger
NF‐*κ*B: nuclear factor‐*κ*B
NFATs: nuclear factor of activated T cells
NT‐proBNP: *N*‐terminal pro‐B‐type natriuretic peptide
PARP‐1: Poly (ADP‐Ribose) Polymerase‐1
PPAR: peroxisome proliferator–activated receptor
PPG: photoplethysmography
RAGE: receptor for AGEs
ROS: reactive oxygen species
RyR2: Ryanodine Receptor Type 2
SCFAs: short‐chain fatty acids
SERCA: sarco/endoplasmic reticulum calcium ATPase
SGLT2: Sodium–Glucose Cotransporter‐2
SNP: single‐nucleotide polymorphism
SOD1: Superoxide Dismutase 1
SOLAECE: Sociedad Latino Americana de Estimulacion Cardiaca y Electrofisiologia
SR: sarcoplasmic reticulum
SVM: support vector machine
TASK‐1: TWIK‐Related Acid‐Sensitive Potassium Channel 1
TGF‐*β*
: transforming growth factor‐beta
TMAO: trimethylamine N‐oxide
TMZ: trimetazidine
TnC: troponin C
TNF: tumor necrosis factor
TnI: troponin I
TnT: troponin T
VEGF: vascular endothelial growth factor

## Author Contributions

**Helin Yang:** writing – original draft, visualization, methodology. **Bing Liang:** writing – review and editing, supervision, conceptualization. **Kexiao Yu:** review and editing.

## Funding

This study was funded by the Top‐notch Young Talent Project of Chongqing Traditional Chinese Medicine Hospital, CQSZYY2020008.

## Ethics Statement

The authors have nothing to report.

## Consent

The authors have nothing to report.

## Conflicts of Interest

The authors declare no conflicts of interest.

## Data Availability

No data were used to support this study.
